# CRISPR/dCas13(Rx) Derived RNA N^6^‐methyladenosine (m^6^A) Dynamic Modification in Plant

**DOI:** 10.1002/advs.202401118

**Published:** 2024-09-04

**Authors:** Lu Yu, Muna Alariqi, Baoqi Li, Amjad Hussain, Huifang Zhou, Qiongqiong Wang, Fuqiu Wang, Guanying Wang, Xiangqian Zhu, Fengjiao Hui, Xiyan Yang, Xinhui Nie, Xianlong Zhang, Shuangxia Jin

**Affiliations:** ^1^ National Key Laboratory of Crop Genetic Improvement Huazhong Agricultural University Wuhan 430070 China; ^2^ Key Laboratory of Oasis Eco‐agricultural Xinjiang Production and Construction Corps/Agricultural College Shihezi University Shihezi 832003 China

**Keywords:** cotton plants, CRISPR/dCas13(Rx), drought tolerance, RNA m^6^A modifications

## Abstract

N^6^‐methyladenosine (m^6^A) is the most prevalent internal modification of mRNA and plays an important role in regulating plant growth. However, there is still a lack of effective tools to precisely modify m^6^A sites of individual transcripts in plants. Here, programmable m^6^A editing tools are developed by combining CRISPR/dCas13(Rx) with the methyltransferase *GhMTA* (**T**argeted RNA **M**ethylation **E**ditor, **TME**) or the demethyltransferase *GhALKBH10* (**T**argeted RNA **D**emethylation **E**ditor, **TDE**). These editors enable efficient deposition or removal of m^6^A modifications at targeted sites of endo‐transcripts *GhECA1* and *GhDi19* within a broad editing window ranging from 0 to 46 nt. TDE editor significantly decreases m^6^A levels by 24%–76%, while the TME editor increases m^6^A enrichment, ranging from 1.37‐ to 2.51‐fold. Furthermore, installation and removal of m^6^A modifications play opposing roles in regulating *GhECA1* and *GhDi19* mRNA transcripts, which may be attributed to the fact that their m^6^A sites are located in different regions of the genes. Most importantly, targeting the *GhDi19* transcript with TME editor plants results in a significant increase in root length and enhanced drought resistance. Collectively, these m^6^A editors can be applied to study the function of specific m^6^A modifications and have the potential for future applications in crop improvement.

## Graphical abstract

TDE and TME editors can efficiently deposit and remove the m^6^A modification at targeted sites, thereby altering mRNA abundance and potentially influencing downstream phenotypes in individual transcripts. These m^6^A editors operate across a broad editing window and exhibit high specificity for off‐target effects, which is essential for investigating the roles of specific m^6^A modifications in plants.

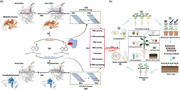

## 1 Introduction

As a basic component of all living organisms, RNA transmits genetic information from DNA to proteins, and various modifications of RNA transcripts occur during this process. RNA modification, collectively known as epitranscriptomics, offers a complex and dynamic level of regulation that ultimately determines cell fate and function, which is reflected in alterations in RNA levels and translation. Since the 1950s, >160 modifications have been discovered to play critical roles in eukaryotic RNA epitranscriptomic pathways, such as N^6^‐methyladenosine (m^6^A), 5‐methylcytosine (m^5^C), and N^1^‐methyladenosine (m^1^A). Among them, m^6^A is the most abundant chemical modification found in eukaryotic mRNA, and it also exists in a variety of bacterial and RNA virus genomes.^[^ ^1^ ^]^ Current studies have demonstrated that m^6^A is involved in several mRNA metabolic processes to regulate RNA fate, and serves as one of the primary mechanisms in RNA post‐transcriptional regulation. These processes include alternative splicing,^[^ ^2^ ^]^ nuclear–cytoplasmic export,^[^ ^3^, ^4^, ^5^ ^]^ RNA stability,^[^ ^6^, ^7^, ^8^ ^]^ 3′‐untranslated regions processing (3′ UTR),^[^ ^9^, ^10^ ^]^ chromatin regulation,^[^ ^11^, ^12^ ^]^ and translation efficiency.^[^ ^13^, ^14^ ^]^ In recent years, research related to m^6^A modifications in plants has increased. These studies have shown that m^6^A modifications play a crucial and extensive role in regulating plant development,^[^ ^15^, ^16^, ^17^, ^18^ ^]^ fruit ripening,^[^ ^19^ ^]^ photomorphogenesis,^[^ ^20^ ^]^ and stress tolerance.^[^ ^21^, ^22^, ^23^, ^24^ ^]^ The m^6^A methylation occurs in a conserved sequence context known as the motif RRACH (R = A/G; H = A/C/U), and other major motifs such as the plant‐specific motif URUAY (Y = C/U).^[^ ^12^, ^13^, ^19^, ^20^, ^25^, ^26^ ^]^ Most previous studies have shown that m^6^A sites are enriched around the stop codon and the 3′ UTR of genes in mammals and plants. Significantly, a substantial number of m^6^A peaks were observed in stress‐responsive transcripts, with enrichment in the 5′ UTR or exon regions of genes.^[^ ^26^, ^27^, ^28^, ^29^ ^]^ It remains uncertain whether the dynamic m^6^A modification in various regions, such as the 3′ UTR or 5′ UTR, follows a similar mechanism for post‐transcriptional regulation.

RNA m^6^A is a dynamic and reversible modification, and the machinery comprises three core subunits, including methyltransferase‐“**writers**”, demethylase‐“**erasers**”, and binding protein‐“**readers**”. In mammals, **writers** are protein complexes that include methyltransferase‐like 3 (METTL3), methyltransferase‐like 14 (METTL14),^[^ ^30^ ^]^ and Wilms tumor 1‐associated protein (WTAP).^[^ ^31^ ^]^ A previous study revealed that METTL3 or a complex of truncated METTL3 and METTL14 possesses methyltransferase activity to methylate adenine in mammals,^[^ ^32^ ^]^ while the in vitro methylation assay has shown that only METTL3, not METTL14, has catalytic activity.^[^ ^33^ ^]^ FTO and ALKBH5 working as **erasers** are responsible for removing m^6^A modifications.^[^ ^34^ ^]^ Different kinds of m^6^A recognition proteins have been identified in mammals, such as YTH (YT521‐B) domain family proteins, and insulin‐like growth factor 2 mRNA binding proteins (IGF2BPs).^[^ ^14^, ^35^ ^]^ In plants, two types of proteins have been identified: the multiprotein writer complex and FIONA1 (a homolog of human METTL16).^[^ ^36^ ^]^ These proteins function as **writers,** responsible for installing m^6^A modifications in mRNA. However, the substrates of FIONA1 are yet to be fully clarified. The multiprotein writer complex includes MTA (a homolog of human METTL3), MTB (a homolog of human METTL14), FIP37 (a homolog of human WTAP), and VIRILIZER (a homolog of human VIRMA).^[^ ^16^, ^19^, ^37^, ^38^, ^39^ ^]^ In terms of **erasers**, there are 13 ALKBH‐homologous proteins, and five of them (ALKBH9A/9B/9C/10A/10B) are homologous to ALKBH5 in *Arabidopsis*.^[^ ^40^ ^]^ It is noteworthy that FTO does not have a homologous counterpart in plants. To date, the demethylase activities of ALKBH9B and ALKBH10B have been demonstrated in plants. Further studies have confirmed that ALKBH10B can function as a bona fide m^6^A demethylase.^[^ ^29^, ^34^, ^41^, ^42^ ^]^ In addition, SlALKBH2 has also been identified as an RNA m^6^A demethylase that is localized to the endoplasmic reticulum in tomato.^[^ ^18^ ^]^ Research on m^6^A readers in plants is limited, mainly focusing on the YTH domain proteins.

Usually, investigations into the impact of m^6^A on various biological processes and phenotypes have relied on the global manipulation of m^6^A **writers**, **erasers,** and **readers** due to the lack of RNA biological tools in plants.^[^ ^39^, ^43^ ^]^ However, the addition or deletion of any key components of the m^6^A modification system resulted in bulk, non‐specific changes in the overall levels of m^6^A modification with unpredictable effects.^[^ ^44^ ^]^ Furthermore, the process of m^6^A demethylation (methylation) at specific regions of individual transcripts may have distinct effects, and the causal relationships between specific m^6^A modifications and downstream phenotypic changes are still unclear. Hence, it is essential to develop a versatile m^6^A editing platform that does not alter the underlying genetic sequence or the overall extent of m^6^A modification. This is vital for studying the significance of individual m^6^A modification sites and understanding the effects of specific RNA methylation events in plants.

Epigenomic editing offers a novel approach to directly investigate the functional implications of epigenomic modifications.^[^ ^45^ ^]^ This technique involves the fusion of epigenetic enzymes, such as **writers** or **erasers**, with CRISPR/dCas9 (dead Cas9), to induce targeted epigenetic modifications at specific DNA sites.^[^ ^46^, ^47^, ^48^ ^]^ CRISPR/Cas13 has been demonstrated to enable precise RNA editing and RNA modification in biological processes, including subtypes VI‐A (Cas13a, 1250 aa), VI‐B (Cas13b, 1150 aa), VI‐C (Cas13c, 1120 aa), VI‐D (Cas13Rx, 930 aa), VI‐X (Cas13X, 790 aa), and VI‐Y (Cas13Y, 792 aa).^[^ ^49^, ^50^, ^51^, ^52^, ^53^, ^54^ ^]^ In contrast to Cas9‐mediated DNA editing technology, the Cas13 protein is distinguished by its smaller physical dimensions and a broader range of protospacer adjacent motifs (PAM). Cas13 has the capability to selectively and reversibly modify RNA without inducing alterations to the genome. Recently, several studies have successfully developed programmable RNA m^6^A or m^1^A methylation or demethylation systems using CRISPR/dCas13 (dead Cas13) in bacteria, mammalian cells, and *Arabidopsis*.^[^ ^32^, ^55^, ^56^, ^57^, ^58^, ^59^, ^60^, ^61^ ^]^ It is important to highlight that some findings from animal studies may not be readily applicable to plant systems. The occurrence of low‐probability and unstable gene editing events is considered incongruent for plant species with more complex genomes. Additionally, exogenous demethylases have significant effects on plant development.^[^ ^62^ ^]^ Presently, there is still a lack of dynamically reversible methylation or demethylation editors in plants, and there are currently no reports of the precise manipulation of individual m^6^A levels for phenotypic studies at the organism level. Most importantly, it remains uncertain whether m^6^A modifications occurring in the 3′ UTR, CDS, and 5′ UTR regions of different transcripts exhibit comparable post‐transcriptional regulatory patterns and how they can regulate transcript metabolism.

In this study, we developed and optimized reversible m^6^A editors by combining CRISPR/dCas13(Rx) with demethyltransferases *GhALKBH* (**T**argeted RNA **D**emethylation **E**ditor, **TDE**) or methyltransferases *GhMTA* (**T**argeted RNA **M**ethylation Editor, **TME**). These m^6^A editors were constructed in plants, making it possible to efficiently modify specific m^6^A sites within various endo‐transcripts in cotton plants. These editors allowed for the successful deposition or removal of the m^6^A modification at targeted m^6^A sites of *GhECA1* and *GhDi19* transcripts within a broad editing window ranging from 0 to 46 nt. Targeting the m^6^A peak sites located in the 3′ UTR of *GhECA1* and the 5′ UTR of *GhDi19* can alter their m^6^A enrichment levels, leading to opposite post‐transcriptional regulation of the mRNAs. Moreover, we found that transgenic plants of TME‐edited *GhDi19* exhibited a substantial increase in root length and an enhancement in drought tolerance. These results suggest that these m^6^A editors could be applied to explore the function of specific m^6^A modifications and have potential applications for future crop improvement.

## 2 Results

### 2.1 Designing and Engineering the Programmable RNA Methylation and Demethylation Tools for Planta Expression

An ideal targeted RNA methylation tool maximizes the likelihood of m^6^A installation, occurring at the target site(s) rather than at the vast excess of nontarget adenines in the transcriptome. The CRISPR/Cas13(Rx) system from *Ruminococcus flavefaciens* was chosen as the anchor protein due to its superior specificity and efficiency.^[^ ^51^, ^53^ ^]^ We conducted codon optimization of the Cas13(Rx) protein based on cotton genome characteristics and optimized the catalytically inactive dCas13(Rx) by mutating R239A, H244A, R858A, and H863A in the conserved RNA‐cleaving HEPN domains.

**Figure 1:**
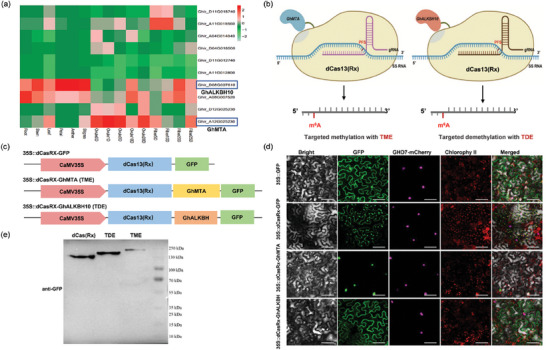
Designing of programmable m^6^A editing tools in plants. a) Tissue expression analysis of homologous genes *GhALKBH9*, *GhALKBH10*, and *GhMTA* in cotton. b) Proposed strategy for the m^6^A methylation (TME) and m^6^A demethylation (TDE) tools. c) Schematic representation of the TME and TDE constructions for tobacco. d) Subcellular localization of dCas13(Rx), TME, and TDE fusion proteins in tobacco leaf cells. 35S::GFP was used as a control. Bar, 25 µm. e) Western blot analysis of dCas13(Rx), TME, and TDE fusion proteins expressed in tobacco leaf cells using an anti‐GFP antibody. A GFP‐tag fused to the C‐terminus of dCas13(Rx) served as a positive control.

To provide useful tools for m^6^A modification, we need to select suitable m^6^A writers or erasers to combine with dCas13(Rx). Our previous study has confirmed that *GhALKBH9* and *GhALKBH10* are essential demethyltransferases for reducing m^6^A levels in cotton.^[^ ^29^ ^]^ Moreover, MTA has been reported and characterized as a key factor that can positively increase m^6^A methylation levels in *Arabidopsis* and *Malus domestica Borkh*.^[^ ^16^, ^26^ ^]^ Subsequently, *GhMTA* gene was identified in cotton genome through homologous comparison. We collected and analyzed the tissue transcript levels of homologous genes *GhALKBH9*, *GhALKBH10*, and *GhMTA* from the reported gene expression profile of cotton.^[^ ^63^ ^]^ Consequently, *GhALKBH10B* (Ghir_D08G007610) and *GhMTA* (Ghir_A12G025230) were selected as the most suitable demethyltransferase and methyltransferase due to their higher transcript levels in most tissues compared to others (**Figure** **1**a). The C‐terminus of dCas13(Rx) was fused to *GhMTA* (dCas13(Rx)‐*GhMTA*, referred to as **TME** editor) and *GhALKBH10* (dCas13(Rx)‐*GhALKBH10*, referred to as **TDE** editor) using flexible linkers to create the programmable m^6^A editors, respectively (Figure 1b).

To verify the subcellular localization of TME and TDE editors, we added a GFP tag at the C‐terminus of the editors and transiently expressed them in tobacco leaves (Figure 1c). The TME‐GFP fusion was localized in the nucleus, while the TDE‐GFP fusion was localized in both the cell membrane and cytoplasm revealed by confocal microscopy (Figure 1d). Western blot analysis revealed successful expression of both TDE and TME fusions in plant cells, as well as the positive control dCas13(Rx) (Figure 1e). These results demonstrate that m^6^A editors TDE and TME were successfully expressed and localized in specific regions of plant cells.

### 2.2 Selection of Targeting Transcripts and Design of the gRNAs

**Figure 2:**
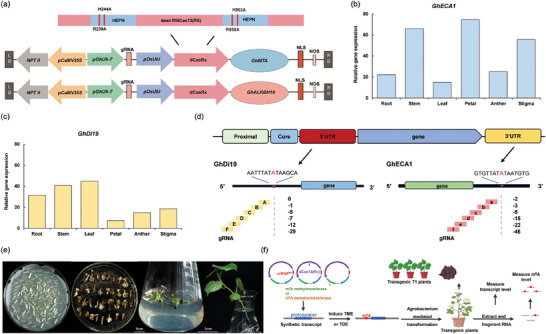
Validation of the programmable m^6^A editing tools in plants. a) Schematic representation of m^6^A methylation (TME) and m^6^A demethylation (TDE) tools for cotton. b) Tissue‐specific expression levels of *GhECA1*. c) Tissue‐specific expression levels of *GhDi19*. d) Schematic representation of specific m^6^A positions on *GhECA1* and *GhDi19* transcripts, along with the regions targeted by a panel of gRNAs. Red letter A indicates the specific m^6^A site located in targeted transcripts. e) *Agrobacterium*‐mediated genetic transformation of cotton using JIN668 as the receptor material. Bars, 1 cm. f) The technical pipeline of programmable m^6^A editing in cotton.

Ectopic overexpression plants driven by the CaMV35S promoter usually exhibit varying disparate expression levels due to the co‐ repression. Therefore, we utilized the ubiquitin promoter from rice to drive the TDE and TME editors, ensuring uniform expression in transgenic plants. To test the site‐specific m^6^A deposition and erasure by TME and TDE editors on individual transcripts in plants, we designed a construct in which dCas13(Rx) is driven by the pOsUbi promoter, and a nuclear localization signal (NLS) is incorporated at the C‐terminus of both TME and TDE. Moreover, the gRNA transcription unit was driven by the endogenous cotton promoter pGhU6‐7 (**Figure** **2**a), which has been demonstrated to be highly efficient in our recent publications.^[^ ^64^, ^65^, ^66^, ^67^, ^68^, ^69^ ^]^

In order to evaluate the site‐specific addition and removal of m^6^A modifications by TME and TDE editors, we aimed to focus on functional genes with known m^6^A modifications. Our previous study constructed an m^6^A modification profile of drought‐sensitive and drought‐resistant cotton at the whole transcriptome level.^[^ ^29^ ^]^ Potential target transcripts were selected according to the following criteria: (1) the RPKM (fragments per kilobase of transcript per million fragments mapped) of a transcript is >300 in m^6^A‐seq. (2) only one m^6^A peak site was identified in the specific region of the target transcript. Therefore, *GhECA1* (Calcium‐transporting ATPase 1), with a single m^6^A site located in the 3′ UTR, was selected as the first targeted transcript, which has been characterized as a key gene in the Ca^2+^ signaling pathway in response to drought stress. Furthermore, *GhDi19* (a drought‐inducible protein), which has a specifical m^6^A peak site located in its 5′ UTR, was selected as another target transcript to further assess the effectiveness of m^6^A editors and explore post‐transcriptional regulatory patterns. A single‐base elongation‐ and ligation‐based qPCR amplification method (known as “SELECT”) further confirmed the presence of the m^6^A site in targeted regions, while the nearby nucleotide did not exhibit any m^6^A modification (Figure S1a,b, Supporting Information). The sequences of the corresponding transcripts were listed in the Supplemental Sequences, and the “A” of the specific m^6^A site in *GhDi19* and *GhECA1* transcripts was marked in red font. Analysis of tissue transcript levels revealed that *GhECA1* exhibits elevated transcript levels in petals, stems, and stigma (Figure 2b), while *GhDi1*9 demonstrates heightened transcript levels in leaves, stems, and roots (Figure 2c).

To investigate editing windows and the efficiency of m^6^A editors TDE and TME, six guide RNAs (gRNAs) were designed to target sequences in the 5′ UTR of *GhDi19* (gRNAs A‐F, positioned at distances of 0, 1, 5, 7, 12, and 29 nt to the nearest m^6^A motif site) and the 3′ UTR of *GhECA1* (gRNAs a‐f, positioned at distances of 2, 3, 5, 15, 22, and 46 nt to the nearest m^6^A motif site), respectively (Figure 2d; Table S1, Supporting Information). *Agrobacterium*‐mediated transformation was used to introduce transgenic plants containing the TME (or TDE) editor and gRNA units (Figure 2e), and the technical process is illustrated in Figure 2f.

### 2.3 Steady Expression of TDE and TME Editors in Transgenic Cotton Plants

**Figure 3:**
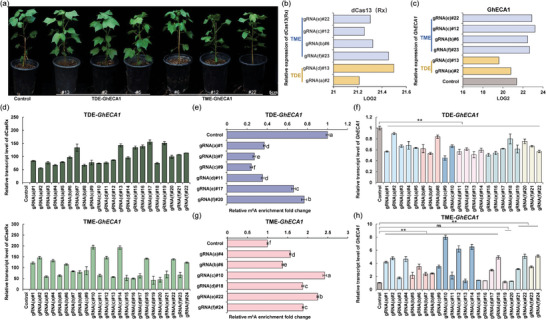
Validation of the programmable TME and TDE m^6^A editors with *GhECA1* transcripts in cotton. a) Phenotypes of transgenic plants with m^6^A‐edited *GhECA1*. Bars, 5 cm. b) Relative expression levels of dCas13(Rx) protein in T0‐positive plants using proteomics. c) Relative expression levels of *GhECA1* using proteomics. d) Relative transcript levels of *dCas13(Rx)* gene in TDE‐edited *GhECA1* plants and TME‐edited *GhECA1* plants. e) Relative m^6^A enrichment; f) Relative transcript levels of *GhECA1* in TDE‐edited T0 plants with a panel of gRNAs at the same stage. g) Relative m^6^A enrichment; h) Relative transcript levels of *GhECA1* in TME‐edited T0 plants with a panel of gRNAs at the same stage. (d‐h) Error bars are presented as the mean ± S.D. (*n =* 3). Statistical significance is indicated by different letters with *p <* 0.01 using Student's t‐test. **p < 0.01.

Based on *Agrobacterium*‐mediated transformation, a large number of transgenic plants were generated and >50 independent T0 plants targeting the *GhECA1* transcript separately containing TME and TDE editors were identified (Table S2, Supporting Information). No obvious phenotypic changes were observed in transgenic plants (**Figure** **3**a). A cohort of m^6^A‐edited *GhECA1* plants was selected for the Parallel Reaction Monitoring (PRM) method to assess the expression of the dCas13(Rx) protein (Figure S1c, Supporting Information). These results demonstrated robust expression of the dCas13(Rx) protein in all selected plants (Figure 3b). Moreover, the targeted transcript *GhECA1* showed decreased expression in TDE‐edited *GhECA1* plants compared to the control, while the expression of *GhECA1* was up‐regulated in TME‐edited *GhECA1* plants (Figure 3c). We proposed that there may be a causal relationship between altered levels of specific m^6^A modifications and downstream mRNA abundance.

### 2.4 Validation of the m^6^A Alterations in the 3′ UTR of the *GhECA1* Transcript of Cotton

More than 25 T0 plants with TDE‐edited *GhECA1* and >30 T0 plants with TME‐edited *GhECA1* were used for transcript‐level analysis. The result showed that the *dCas13(Rx)* gene was well transcribed in these transgenic plants as detected by RT‐qPCR. However, there was some variation among the different edited monocots (Figure 3d). Traditional methods for assessing site‐specific m^6^A modification, such as MeRiP–qPCR (Methylated RNA immunoprecipitation coupled with RT–qPCR) or sequencing‐based approaches like miCLIP (methylation‐individual‐nucleotide resolution cross‐linking and immunoprecipitation), lack either single‐nucleotide resolution or quantitative capability. Therefore, the SELECT method was used to identify and quantify m^6^A methylation levels of targeted transcripts in plant cells, which can measure m^6^A levels with single‐nucleotide resolution through qPCR.^[^ ^70^ ^]^

Compared to the control, TDE‐edited *GhECA1* plants with six gRNAs (a‐f) significantly reduced m^6^A levels by 62.8 ± 1.47% in gRNA(a)#1, 72.7 ± 1.36% in gRNA(b)#7, 75.7 ± 1.35% in gRNA(c)#9, 64.5 ± 1.44% in gRNA(d)#11, 33.8 ± 1.88% in gRNA(e)#17, and 23.2 ± 2.65% in gRNA(f)#20, respectively (Figure 3e). The transcript levels of *GhECA1* decreased by 43.1%, 46.5%, 54.9%, 43.4%, 37.9%, and 24.4% in the corresponding plants. The most pronounced demethylation and down‐regulation of *GhECA1* were observed in gRNA(c)#9, which targeted the region approximately 5 nt downstream of the m^6^A site. Significantly, the transcript abundance of *GhECA1* consistently decreased across all 22 transgenic T0 plants, with an average transcript level of 30.9%, 33.4%, 44.1%, 42.9%, 38.2%, and 33.6% following gRNAs a‐f compared to the control (Figure 3f).

In TME‐edited *GhECA1* regenerated plants, we found that the m^6^A level significantly increased to 1.57‐fold in gRNA(a)#4, 1.38‐fold in gRNA(b)#6, 2.43‐fold in gRNA(c)#10, 1.88‐fold in gRNA(d)#18, 2.25‐fold in gRNA(e)#22, and 1.89‐fold in gRNA(f)#24, respectively (Figure 3g). The transcript levels of *GhECA1* were measured, showing an increase of 4.64‐, 3.50‐, 7.98‐, 4.91‐, 5.07‐, and 5.11‐fold in the corresponding plants. Notably, the highest increase in the m^6^A level and transcript level reached up to 7.98‐fold in gRNA(c)#10 (Figure 3h). Upon analyzing transcript levels in all TME‐edited *GhECA1* plants, a notable increase in the transcript level of *GhECA1* was observed. The average transcript levels were markedly increased to 3.84‐, 2.63‐, 4.15‐, 2.64‐, 3.15‐, and 3.43‐fold following gRNAs a‐f, although the editing effect showed variability among the different gRNAs target sites. Control plants showed no significant alteration in m^6^A levels at the targeted sites. These results demonstrate that both TME and TDE editors can significantly alter the m^6^A levels of specific sites and regulate the transcript levels of target transcripts. All gRNAs can achieve effective editing, demonstrating the flexible and broad editing window of the TME and TDE editors.

### 2.5 Targeting m^6^A Modification of *GhECA1* with TME Editor Enhanced Drought Tolerance in Cotton Plants

**Figure 4:**
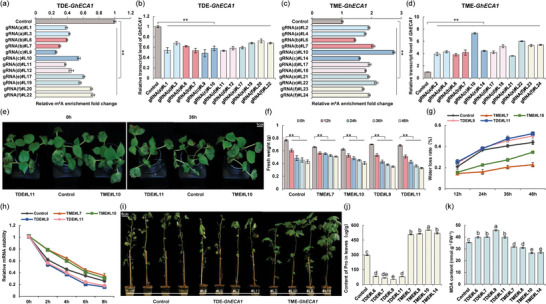
The m^6^A levels of *GhECA1* transcripts altered by programmable m^6^A editors were faithfully inherited by T1 cotton progeny. a) Relative m^6^A enrichment; b) Relative transcript levels of *GhECA1* in TDE‐edited T1 plants at the same stage. c) Relative m^6^A enrichment; d) Relative transcript levels of *GhECA1* in TME‐edited T1 plants at the same stage. (a‐d) Error bars are given as the mean ± S.D. (*n =* 3). **p < 0.01. e) Leaf phenotypes; Bars, 1 cm. f) Fresh weight; **p < 0.01. g) The water loss rate of 4‐week‐old T1 seedlings treated with 15% PEG‐6000 for 48 h. h) The mRNA lifetimes of *GhECA1* transcripts in 4‐week‐old plants were measured after transcription inhibition (TI). Error bars are given as the mean ± S.D. (*n =* 3). i) Phenotypes; Bars, 5 cm. j) MDA content; k) Pro content in leaves of 10‐week‐old T1 plants after 7 days of drought. Error bars are given as the mean ± S.D. (*n =* 3). Statistical significance is indicated by different letters with *p <* 0.01 using Student's t‐test.

The genetic stability of gene editing tools, particularly in plants with complex genomes that necessitate genetic transformation and regeneration, is crucial for preserving stable editing and targeted traits in subsequent generations. To assess the editing efficiency, heritability, and associated traits of m^6^A editors on targeted transcripts in multi‐generational plants, a significant number of TDE‐edited *GhECA1* and TME‐edited *GhECA1* T1 plants were cultivated at the same stage. Molecular detection and RNA transcript analysis revealed that >12 plants (from various lines) with a consistent transcript level of *dCas13(Rx)* gene for each construct were screened to assess m^6^A levels and drought tolerance (Figure S1d,e, Supporting Information). These results showed that the TDE editor led to a significant decrease in m^6^A levels, with reductions of 61.1% in gRNA(a)#L1, 69.6% in gRNA(b)#L7, 74.3% in gRNA(c)#L9, 62.5% in gRNA(d)#L11, 39.2% in gRNA(e)#L17, and 29.5% in gRNA(f)#L20 compared to the control (**Figure** **4**a). The transcript levels of *GhECA1* decreased by 45.9%, 46.5%, 51.7%, 46.7%, 40.8%, and 27.7% in the corresponding plants. All TDE‐edited *GhECA1* plants exhibited a decrease in the transcript levels of *GhECA1* gene, with downregulation ranging from 48.3% to 85.9% (Figure 4b).

Next, we evaluated the “writing” efficiency of the TME editor in T1 plants and found that the m^6^A enrichment was significantly increased by 1.79‐fold in gRNA(a)#L4, 1.44‐fold in gRNA(b)#L6, 2.75‐fold in gRNA(c)#L10, 1.80‐fold in gRNA(d)#L18, 2.15‐fold in gRNA(e)#L22, and 1.92‐fold in gRNA(f)#L24 (Figure 4c). In the corresponding T1 plants, the transcript levels of *GhECA1* increased significantly by 4.30‐, 3.82‐, 7.32‐, 5.20‐, 6.03‐, and 5.43‐fold, respectively. The transcript levels in all tested plants showed an upregulation range of 3.63‐fold to 7.32‐fold (Figure 4d). Targeting of gRNA sites has been found to impact the efficiency of m^6^A editors and regulate the expression of targeted transcripts (Figure S1f,g, Supporting Information). These findings indicate a positive correlation between increased methylation at the target sites within the 3′ UTR of the *GhECA1* transcript and gene transcript levels. Furthermore, these results suggest that transgenic *GhECA1* plants exhibit typical m^6^A modification editing, which can be reliably inherited from T0 plants to T1 progeny.

Four‐week‐old TDE‐*GhECA1*‐gRNA(c)#L9 (named TDE#L9), TDE‐*GhECA1*‐gRNA(d)#L11 (named TDE#L11), TME‐*GhECA1*‐gRNA(b)#L7 (named TME#L7), and TME‐*GhECA1*‐gRNA(c)#L10 (named TME#L10) T1 seedlings were selected (six seedlings per line) and divided into six groups each. The 15% polyethylene glycol‐6000 (PEG‐6000) solution was used to simulate drought stress for these T1 seedlings. After 36 h of drought treatment, TDE#L9 and TDE#L11 lines exhibited drought‐sensitive phenotypes compared to the wild‐type seedlings, whereas TME#L7 and TME#L10 lines displayed drought‐resistant phenotypes. As shown, seedlings of the TDE#L11 line exhibited a more severe leaf wilt phenotype compared to the control seedlings, whereas the TME#L10 line did not show obvious wilting symptoms (Figure 4e). Malondialdehyde (MDA) content in the leaves and roots of TME#L10 seedlings was significantly lower than that in TDE#L11 and control after drought treatment, indicating that TME‐edited plants experienced a lower degree of stress injury (Figure S1h,i, Supporting Information). After 48 h of drought treatment, the decrease in fresh weight of TME#L7 and TME#L10 lines was significantly lower than that of TDE#L9, TDE#L11, and wild‐type plants (Figure 4f). Meanwhile, the water loss rate in the TME‐edited lines was also significantly lower than that of the wild type, while that of the TDE‐edited lines was significantly higher than that of the wild type (Figure 4g).

Most importantly, the transcript levels of *GhECA1* in the leaves of TME‐edited lines were significantly elevated compared to seedlings without PEG treatment, surpassing that of control plants as well as TDE‐edited lines at the same stage (Figure S2a, Supporting Information). To investigate whether m^6^A modifications of *GhECA1* could alter mRNA stability and subsequently affect mRNA abundance through post‐transcriptional regulation, we performed transcription inhibition (TI) assays using actinomycin D to evaluate the lifetimes of *GhECA1* transcripts in leaves. We found that the degradation rate of the *GhECA1* transcript in TDE‐edited plants was higher compared to the control, while that of TME‐edited plants was comparatively lower than that of the control (Figure 4h). These results indicate that reducing m^6^A levels of *GhECA1* by TDE editor can promote transcript degradation while increasing m^6^A levels of *GhECA1* by TME editor can inhibit transcript degradation. These data validate the ability of both editors to accurately introduce or remove methylation modifications from targeted transcripts, thereby influencing gene transcript levels by modulating mRNA stability.

Furthermore, 10‐week‐old T1 transgenic plants from various lines (three plants per line) were selected for direct exposure to drought stress in small pots. The relative water content of these plants before drought treatment was randomly examined, and the result indicated that TME‐edited plants had higher water content compared to wild‐type plants and TDE‐edited plants (Figure S2b, Supporting Information). After 7 days of drought treatment, TDE‐edited and wild‐type plants showed significant wilting, while TME‐edited plants exhibited overall healthy growth with less leaf shedding (Figure 4i). The proline (Pro) content in TME‐edited plants was significantly higher (Figure 4j), while the MDA content was significantly lower than that of wild‐type and TDE‐edited plants at this time (Figure 4k). These results indicate that TME and TDE can alter the transcript level of *GhECA1* and influence the drought tolerance of edited plants.

### 2.6 Validation of the m^6^A Editors in the 5′ UTR of the *GhDi19* Transcripts of Cotton

**Figure 5:**
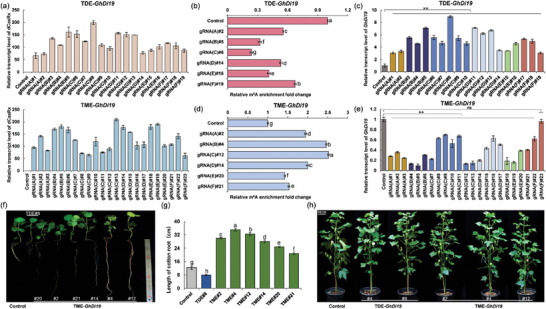
Validation of the programmable TDE editors targeting *GhDi19* transcript to promote root growth in cotton. a) Relative transcript levels of *dCas13(Rx)* gene in m^6^A‐edited T0 plants. b) Relative m^6^A enrichment; c) Relative transcript levels of *GhDi19* in TDE‐edited plants at the same stage. d) Relative m^6^A enrichment level; e) Relative transcript levels of *GhDi19* in TME‐edited T0 plants at the same stage. (a‐e) Error bars are given as the mean ± S.D. (*n =* 3). Statistical significance is denoted by different letters with *p <* 0.05 using Student's t‐test. **p < 0.01. f) Root phenotypes; Bars, 1 cm. g) Root length of T0 transgenic plants. Error bars are given as the mean ± S.D. (*n =* 3). Statistical significance is indicated by different letters with *p <* 0.05 using Student's t‐test. h) Phenotypes of T1 cotton plants with m^6^A‐edited *GhECA1* in the adult stage. Bars, 10 cm.

In order to assess the potential of m^6^A editors for precise targeting and editing of various transcripts, we conducted an analysis of the changes in m^6^A methylation levels of *GhDi19* to further explore the correlation between methylation modifications and gene expression in plants. More than 25 T0 plants of TDE‐edited *GhDi19* and >30 T0 adult plants of TME‐edited *GhDi19* with high expression of dCas13(Rx) protein were used for further analysis (**Figure** **5**a; Table S2, Supporting Information). SELECT analysis showed that the m^6^A level of TDE‐edited *GhDi19* plants significantly decreased by 44.3 ± 2.41% in gRNA(A)#2, 67.6 ± 1.8% in gRNA(B)#5, 76.2 ± 1.4% in gRNA(C)#8, 46.7 ± 1.5% in gRNA(D)#14, 58.6 ± 1.5% in gRNA(E)#16, and 32.4 ± 1% in gRNA(F)#19, respectively (Figure 5b). This further demonstrates the robust editing efficiency within the broad editing window, spanning 0–29 nt upstream of the targeted sites. Interestingly, the transcript levels of *GhDi19* in TDE‐edited plants exhibited a significant increase from 3.07‐ to 8.93‐fold compared to the control (Figure 5c).

Accordingly, TME‐edited *GhDi19* plants resulted in an increase in m^6^A enrichment of *GhDi19* by 1.95‐fold in gRNA(A)#2, 2.46‐fold in gRNA(B)#4, 2.52‐fold in gRNA(C)#12, 2.0‐fold in gRNA(D)#14, 1.43‐fold in gRNA(E)#20, and 1.53‐fold in gRNA(F)#21 (Figure 5d), followed by a decrease in *GhDi19* mRNA transcript levels (Figure 5e). Taken together, both TDE and TME editors can regulate the m^6^A level of *GhDi19*, which is consistent with previous data on *GhECA1* transcript. However, the modification level of the specific m^6^A site located on the 5′ UTR of the *GhDi19* transcript and the specific site located on the 3′ UTR of the *GhECA1* transcript exhibited an opposite pattern of transcriptional level regulation.

Significantly, the TME editor, targeting the 5′ UTR of *GhDi19* with different gRNAs, was able to induce longer roots in >10 T0 plants at the same stage compared to the control. In contrast, the roots of the TDE‐edited *GhDi19* T0 plants were much shorter (Figure 5f). By measuring the lengths of cotton plant roots, we found that the roots of TME‐edited *GhDi19* plants were significantly longer than those of TDE‐edited *GhDi19* and control plants (Figure 5g). These results are consistent with a previous study that demonstrated an increase in root length in *AtDi19* knockout mutants and a decrease in root length in *AtDi19* overexpression lines compared to the wild type in *Arabidopsis*.^[^ ^71^ ^]^ No obvious phenotypic differences were observed in the above‐ground parts of mature plants, which may be attributed to the tissue‐specific expression pattern of *GhDi19* and its predominant regulatory role in leaves, stems, and roots (Figure 5h). To our knowledge, there have been no reports linking the manipulation of m^6^A levels in specific genes to the phenotype of any species. Our results suggest that manipulating the m^6^A methylation of target transcripts can regulate gene expression, potentially leading to plant phenotypes similar to those achieved through overexpression or knockdown/knockout approaches.

### 2.7 Targeting *GhDi19* with m^6^A Editors Resulted in Drought Resistant in T1 Progeny

**Figure 6:**
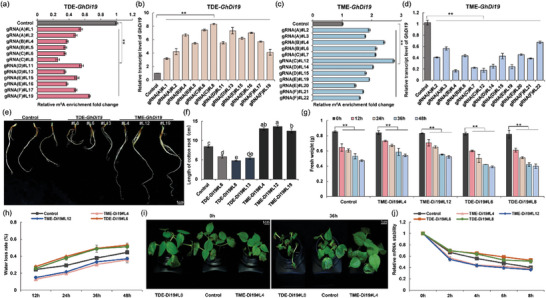
Targeting *GhDi19* modification with programmable m^6^A editors induces root growth resulting in drought tolerance in cotton. a) Relative m^6^A enrichment; b) Relative transcript levels of *GhDi19* in TDE‐edited T1 plants at the same stage. c) Relative m^6^A enrichment; d) Relative transcript levels of *GhDi19* in TME‐edited T1 plants at the same stage. (a‐d) Mean ± S.D. (*n =* 3). **p < 0.01. e) Root phenotypes; Bars, 1 cm. f) Root length of m^6^A‐edited seedlings at the 4‐week‐old stage. Mean ± S.D. (*n =* 3). Statistical significance is indicated by different letters with *p <* 0.05 using Student's t‐test. g) Fresh weight; **p < 0.01. h) The water loss rate of 4‐week‐old T1 seedlings treated with 15% PEG‐6000 for 48 h. i) Phenotypes of 4‐week‐old T1 seedlings treated with 15% PEG‐6000 for 36 h. Bars, 1 cm. j) The mRNA lifetimes of *GhDi19* transcripts in 4‐week‐old plants after transcription inhibition (TI). Error bars are presented as the mean ± S.D. (*n =* 3).

Several T1 plants of TDE‐edited *GhDi19* and TME‐edited *GhDi19* were cultivated to the same stage. More than 14 positive plants were selected from each editor to detect the m^6^A enrichment and transcript levels. RNA transcript analysis confirmed the expression level of the dCas13(Rx) protein (Figure S2c,d, Supporting Information). SELECT showed that the m^6^A methylation levels of TDE‐edited *GhDi19* decreased by 25.7% to 65.7% compared to the control (**Figure** **6**a), followed by an upregulation of *GhDi19* transcript levels ranging from 3.17‐fold to 8.32‐fold (Figure 6b). Additionally, the m^6^A methylation level of TME‐edited *GhDi19* plants increased from 1.44‐fold to 2.75‐fold (Figure 6c), and gene transcript levels were downregulated by 16.9% to 67.5% in transgenic T1 progeny compared to the control (Figure 6d). Similarly, the m^6^A editor uses different gRNAs to achieve editing, and there are variations at the target position of the gRNAs that impact the transcript levels of *GhDi19* (Figure S2e,f, Supporting Information). Phenotype analysis revealed that the roots of TME‐edited *GhDi19* seedlings were significantly longer than those of the control plants, while the roots of TDE‐edited *GhDi19* seedlings were shorter than those of the control plants (Figure 6e). Measurement of root length further demonstrated considerable variation among TME‐edited *GhDi19*, TDE‐edited *GhDi19*, and control plants (Figure 6f).

To confirm the genetic stability of the m^6^A‐edited *GhDi19* plants, 4‐week‐old T1 seedlings of TME‐*GhDi19*‐gRNA(B)#L4 (referred to as TME‐Di19#L4), TME‐*GhDi19*‐gRNA(C)#L12 (referred to as TME‐Di19#L12), TDE‐*GhDi19*‐gRNA(C)#L6 (referred to as TDE‐Di19#L6), and TDE‐*GhDi19*‐gRNA(C)#L8 (referred to as TDE‐Di19#L8) (six seedlings per line) were cultured to the same stage and then subjected to hydroponic treatment with 15% PEG‐6000. After 48 h of PEG‐6000 treatment, TDE‐edited lines exhibited drought‐sensitive phenotypes compared to the wild‐type plants, whereas TME‐edited lines displayed drought‐tolerant phenotypes. The decrease in fresh weight of TME‐Di19#L4 and TME‐Di19#L12 lines was significantly lower compared to that of TDE‐Di19#L6 and TDE‐Di19#L8, and wild‐type plants (Figure 6g). Meanwhile, the water loss rate in the TME‐edited lines was significantly lower than that of the wild type, while that of the TDE‐edited lines was significantly higher than that of the wild type (Figure 6h). The TDE‐Di19#L8 seedlings exhibited a more severe leaf wilt phenotype compared to the control seedlings, whereas the cotyledons and leaves of TME‐Di19#L4 did not show any significant wilting symptoms (Figure 6i). MDA content in the roots of TME‐Di19#L4 seedlings was significantly lower than that in TDE‐Di19#L8 and wild‐type after drought treatment, indicating that TME‐edited plants experienced a lower degree of stress injury (Figure S2g, Supporting Information).

We also conducted transcription inhibition (TI) assays to assess the lifetimes of *GhDi19* transcripts in the leaves of m^6^A‐edited cotton plants. The results showed that the degradation rate of the *GhDi19* transcript in TME‐edited plants was higher compared to the control, while that of TDE‐edited plants was comparatively lower than that of the control (Figure 6j). These results indicate that m^6^A modifications in the 3′ UTR of the *GhDi19* transcript could affect mRNA abundance by influencing mRNA stability.

**Figure 7:**
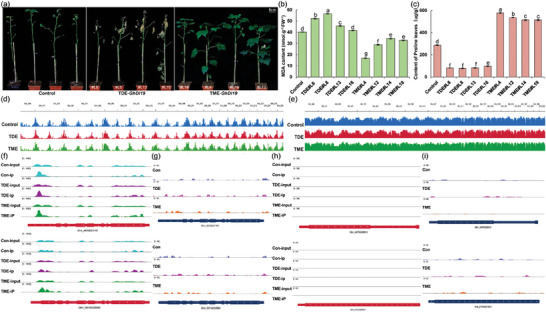
Specificity and potential off‐target effects analysis of TDE and TME editors targeting *GhECA1*. a) Phenotypes; Bars, 5 cm. b) MDA content; c) Pro content in the leaves of 2‐month‐old plants after 7 days of drought. Mean ± S.D. (*n =* 3). Statistical significance is denoted by different letters with *p <* 0.05 using Student's t‐test. d) Integrated genome view of m^6^A peaks; e) Integrated genome view of ATAC‐seq libraries from edited plants of Control, TME, and TDE. Gene tracks are displayed above the MeRIP‐seq and ATAC‐seq tracks. MeRIP‐seq analysis was performed with three independent biological replicates. ATAC‐seq analysis was performed with two independent biological replicates. f) Genome browser view of m^6^A peaks; g) Genome browser tracks of ATAC‐seq libraries in *GhECA1* transcript, with homologous genes located on the At and Dt subgroups, in Control, TME, and TDE plants. h) Genome browser view of m^6^A peak; i) Genome browser tracks of ATAC‐seq libraries on the top 2 transcripts with the highest similarity to the target sequence.

In addition, 8‐week‐old transgenic plants from different lines (three plants per line) were selected for direct exposure to drought stress. The relative water content of these plants before treatment was randomly examined, and the results indicated that TME‐edited plants had higher water content compared to wild‐type plants and TDE‐edited plants (Figure S2h, Supporting Information). After the 7‐day drought treatment, TDE‐edited and wild‐type plants showed significant wilting, while TME‐edited plants exhibited overall healthy growth with less leaf shedding (**Figure** **7**a). Measurement of the MDA content in TDE‐edited cotton plants was significantly higher than in the control and TME‐edited plants (Figure 7b), while the proline content was significantly lower than that of the control and TME‐edited plants (Figure 7c). These results indicate that transgenic cotton plants containing the TDE and TME editors exhibited significant root elongation phenotypes and drought tolerance, further demonstrating the genetic stability of our editors as well as the heritability of the phenotypes.

### 2.8 High Specificity and Undetectable Off‐target Effects were Observed in TME‐ and TDE‐edited Cotton Plants

To investigate the specificity of the editors TDE and TME for site‐specific addition and removal of target transcripts, we employed various methods to assess their potential off‐target effects. First, the 12 designed gRNAs were examined for similarity to other sequences using whole‐transcriptome BLAST. Cotton, a heterotetraploid species, exhibits significant similarity between the sequences of its homologous genes located on the At and Dt subgroups. We found that the gRNAs targeting *GhECA1* had sequences consistent with the homologous genes, but there were at least 7 nucleotide mismatches with non‐target sequences outside the homologous genes. The gRNAs targeting *GhDi19* had a 3‐nt mismatch with the sequence of the homologous gene and at least a 5‐nt mismatch with other non‐target sequences (Supplemental Excel, Supporting Information).

Numerous studies have reported the correlation between chromatin accessibility and gene expression levels.^[^ ^72^, ^73^, ^74^ ^]^ Stable expression of RNA demethylases in plants, such as FTO, can alter chromatin accessibility and impact gene expression.^[^ ^62^, ^75^ ^]^ Therefore, we conducted transcriptome‐wide MeRIP sequencing (MeRIP‐seq), Assay for Transposase‐Accessible Chromatin with high‐throughput sequencing (ATAC‐seq), and RNA sequencing (RNA‐seq) to analyze the overall characterization of m^6^A peaks and chromatin accessibility in control (wild‐type), TME‐*GhECA1*, and TDE‐*GhECA1* plants (Figure S3a–c, Supporting Information). By comparing global m^6^A enrichment and chromatin accessibility, variations were observed in localized chromosomal regions. However, it was found that the levels of m^6^A enrichment and chromatin accessibility in Control, TME‐*GhECA1*, and TDE‐*GhECA1* plants followed a more consistent trend at the whole transcriptome and chromosome levels (Figure 7d,e). Compared to the enriched m^6^A peaks throughout the whole transcriptome, we observed that the majority of the m^6^A peaks were shared between the edited lines and the control. The lower number of differential peaks in m^6^A enrichments indicated minimal off‐target effects in all the edited lines (Figure S4a, Supporting Information). Further analysis revealed only one m^6^A peak in the 3′ UTR region of *GhECA1* transcripts, which was higher in TME‐*GhECA1* than in the control and TDE‐*GhECA1* (Figure 7f). These results further confirm the m^6^A editing capability of our TME and TDE editors on endogenous transcripts. The chromatin accessibility of *GhECA1* transcripts was higher in TDE‐*GhECA1* compared to the control and TME‐*GhECA1* (Figure 7g). Correlation analysis of RNA‐seq and ATAC‐seq data indicated a positive correlation between chromatin accessibility and gene expression (Figure S4b, Supporting Information). Furthermore, we analyzed the m^6^A peak and chromatin accessibility of the top 4 transcripts that showed the highest similarity to the target sequence to evaluate the off‐target effects of TDE and TME editors. There were no m^6^A peaks in the transcripts of Ghir_A07G006510 or Ghir_D10G023150 (Figure 7h), which had 8‐nt mismatches with the highest off‐target score. Additionally, there was no significant change in their chromatin accessibility (Figure 7i). This pattern was also observed in two other transcripts with high off‐target scores (Figure S3d, Supporting Information). These results indicate that TME and TDE editors exhibited specific methylation (demethylation) abilities and limited off‐target effects, possibly due to the specificity of the dCas13Rx protein and the locations of specific m^6^A peaks on various transcripts.

**Figure 8:**
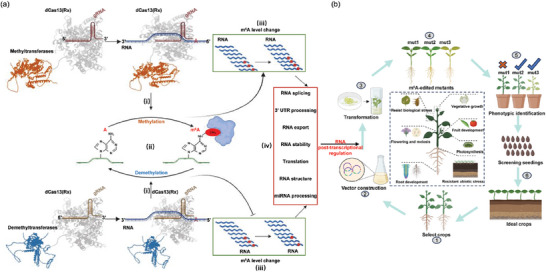
The working pipeline of a programmable m^6^A editing tool for plants. a) Schematic of CRISPR/dCas13(Rx)‐m^6^A unit in the proposed m^6^A editing system. b) The proposed m^6^A editing platform for plants: 1) High‐throughput sequencing to identify m^6^A modifications that may have crucial biological functions during growth, development, or stress resistance in crops. 2) The m^6^A editing system adds or removes specific m^6^A sites of targeted transcripts that may have significant biological functions. 3) *Agrobacterium*‐mediated genetic transformation to generate transgenic plants. 4) Generation of the m^6^A‐edited plants. 5) Phenotyping identification of m^6^A‐edited plants and screening for desired plants. 6) Further analysis of screened plants in a greenhouse or field.

In summary, our m^6^A editing process can be divided into four steps (**Figure** **8**a): i) The establishment of a complete m^6^A editing platform in plants by fusing the CRISPR/dCas13(Rx) with m^6^A demethylation or methylation enzymes. ii) The dCas13(Rx) protein complex is guided to a specific RNA site by gRNA and PFS. The main requirement for this step is to accurately draw dynamic m^6^A modification maps at single‐base resolution, which are used to design the gRNA sequence that guides the dCas13(Rx) complex to a specific site on the mRNA. iii) Writers or erasers fused to the dCas13(Rx) complex can install or remove m^6^A at a specific RNA site. The primary requirement for this process is that the writers and erasers have the ability to add or remove m^6^A sites on RNA in plants. iv) The use of m^6^A editors can modify the specific m^6^A site on target transcripts, subsequently influencing gene expression through RNA splicing, RNA export, 3′ UTR processing, alternative polyadenylation, RNA structure, RNA stability, and translation.

Therefore, we have proposed a working model of the complete m^6^A editing platform to study the function of specific m^6^A modification sites and improve crop yield, quality, and stress resistance (Figure 8b). Initially, we identify specific m^6^A sites on RNA that may have crucial biological functions during plant growth, development, or stress resistance using high‐throughput sequencing at single‐base resolution. Subsequently, m^6^A editors, guided by designed gRNAs, add or remove specific m^6^A modifications to regulate gene expression, including transcriptional and post‐transcriptional regulation. Finally, m^6^A‐edited mutants are obtained, and their phenotypes are identified in multiple generations to screen for the “desired plant”. This step is crucial because the m^6^A‐edited mutants are selected with the expectation of possessing better agronomic traits than the wild type.

## 3 Discussion

RNA modifications are essential components of the epitranscriptome, with highly dynamic and reversible m^6^A modifications regulating nearly all aspects of RNA metabolism and functionality.^[^ ^45^, ^76^ ^]^ m^6^A modification is involved in regulating many important agronomic traits, such as plant vegetative growth,^[^ ^39^, ^77^, ^78^ ^]^ root development,^[^ ^22^ ^]^ and fruit maturity.^[^ ^18^, ^19^ ^]^ Editing m^6^A modifications on important RNA transcripts during growth, development, and photosynthesis may be an effective strategy to enhance crop yield, quality, and stress tolerance. The development of various genome editing tools allows for the functional analysis of epigenomic marks and facilitates the investigation of epigenetic control over biological processes. However, much of the current knowledge about m^6^A is mainly based on genetic perturbations induced by global overexpression or knockout of relevant genes, such as MTA, MTB, and FIP37, which would modify the entire transcriptome rather than specific target sites of interest.^[^ ^16^, ^39^, ^44^ ^]^ To investigate the site‐specific effects of m^6^A interacting with multiple readers, it is essential to have a strategic approach to manipulate targeted m^6^A sites within endo‐transcripts.

In this study, we first developed dCas13(Rx)‐mediated m^6^A editors by combining it with RNA methyltransferase *GhMTA* (TME) or demethylase *GhALKBH10* (TME) to trigger demethylation or methylation of specific m^6^A sites in plants. Both epitranscriptomic editors were localized to the nucleus, enabling site‐specific installation or removal of m^6^A through CRISPR/dCas13 engineering. CRISPR/dCas13 specifically targets RNA and is not restricted by the typical PAM (PFS) limitation, thus expanding its applications in epitranscriptomic editing compared to systems based on the CRISPR/dCas9 system. Additionally, the high mismatch intolerance of gRNAs provides m^6^A editors with greater precision compared to other nucleic acid‐editing tools.^[^ ^51^, ^79^ ^]^ Most importantly, RNA modification‐based therapies could achieve changes in genetic information without altering the genomic sequences, thus eliminating concerns about introducing permanent alterations through DNA targeting.

Both editors successfully achieved precise and efficient bidirectional modulation of specific m^6^A sites in the 3′ UTR of *GhECA1* and 5′ UTR of *GhDi19*. All gRNAs could mediate effective m^6^A editing, exhibiting a broad scope ranging from 0–46 nt of the m^6^A motif sites. This indicates that TME and TDE editors possess great flexibility and precision targeting capabilities for m^6^A editing at specific transcriptome‐specific loci. The broad editing window may be attributed to the editors' flexible accessibility to the m^6^A site, resulting from the complex three‐dimensional structure of target chromatin and the long protein linkers with editor proteins. Additionally, the targeting sites of gRNAs have effects on the editing efficiency. RNA demethylation (methylation) seems not to be influenced by either the 5′ or 3′ sequence of the dCas13(Rx)‐targeted sites. Moreover, we demonstrated that the TME and TDE editors displayed specific methylation or demethylation abilities and limited off‐target effects using MeRIP‐seq and ATAC‐seq. Collectively, these results suggest that our programmable m^6^A editing tools based on the CRISPR/dCas13(Rx) system can be easily applied to study m^6^A modifications of specific genes in plants.

*GhECA1* and *GhDi19* have been identified as key endogenous transcripts for drought response in cotton. Strikingly, we found that m^6^A modification played opposing roles in regulating the mRNA transcript levels of *GhECA1* and *GhDi19*. The TDE editor decreased the transcript level of *GhECA1* but increased the *GhDi19* transcript level, while the TME editor increased the transcript levels of *GhDi19* and decreased the transcript levels of *GhDi19*. This may be due to the fact that m^6^A modification functions can regulate gene expression by controlling RNA fate through various mechanisms in post‐transcriptional RNA regulation (such as alternative splicing, and RNA export) when targeted to different regions (3′ UTR/5′ UTR). However, the regulatory mechanism underlying this process remains unknown and requires further exploration. Our m^6^A editors precisely modified the m^6^A sites in the 3′ UTR of *GhECA1* and the 5′ UTR of *GhDi19* to create valuable cotton germplasms, which can be utilized to explore the mode and mechanism of m^6^A transcriptional regulation in the future. Interestingly, targeting the *GhDi19* transcript with the TME editor significantly induced root growth, resulting in enhanced drought resistance, which can be faithfully transmitted from T0 parental plants to T1 progenies. These results demonstrate that m^6^A editors can be used to clarify previously ambiguous interactions and causal relationships between m^6^A modifications and phenotypes. Targeted demethylation (or methylation) holds the promise of inducing long‐lasting effects on specific targets, providing a programmable and in vivo manipulation tool to edit specific mRNA in the transcriptome.

Several optimizations are still needed for the current TME and TDE editors. A key limitation of CRISPR/Cas technology is that the Cas protein must be precisely localized by gRNA. Therefore, the primary challenge for m^6^A editing in plant systems is to accurately map dynamic m^6^A modifications at single‐base resolution, which has been hindered by the limitations of m^6^A detection methods. Secondly, fewer **erasers** than **writers** have been identified in plants. It is important to identify more m^6^A enzymes for the use of m^6^A editing systems, especially in major crop species. Additionally, we propose utilizing **readers** in combination with the dCas13‐**eraser** fusion protein to enhance the accuracy of m^6^A loci recognition. Furthermore, we recommend employing directed evolution of the effector protein to boost the activity of m^6^A editors. Finally, the implementation of “dual selection” of gRNA and readers may substantially decrease “off‐target” effects and improve the efficiency of removing m^6^A modifications from the target site by the complex.

By coupling CRISPR/dCas13 technology, the m^6^A editors described here offer a versatile toolbox to unlock the secrets of the epitranscriptome. It is possible to achieve site‐specific m^6^A installation or erasure, which is crucial for understanding the localized effects of mRNA methylation. Considering the success of high‐throughput functional genomic screening based on CRISPR/Cas9 technology, a proof‐of‐concept dCasRx‐based strategy that could enable high‐throughput screening of m^6^A modifications in the whole epitranscriptome using a suitable gRNA library holds broad applications in various studies. In addition, the m^6^A editing platform can be used as a model system for developing and editing other RNA molecule modifications, such as 5‐methylcytidine (m^5^C), 1‐methylguanosine (m^1^G), and other unreported RNA modifications in plants. We believe that m^6^A editing will emerge as a crucial tool for investigating the functions of m^6^A modifications, providing a new approach to understanding epigenetic regulation, and enhancing plant agronomic traits for crop quality improvement in the future.

## 4 Experimental Section

### Vector Construction

The codon‐optimized dCas13(Rx) with mutated nuclease domains (R239A/H244A/R858A/H863A) was synthesized by Kingsley Company. The RGEB32‐GhU6.7‐NPT2 vector was linearized using *BstB*I and *Xba*I. Subsequently, the full‐length dCas13(Rx) was ligated into the linearized pRGEB32‐GhU6.7‐NPT2 using the ClonExpress II One Step Cloning Kit to obtain pRGEB32‐GhU6.7‐dCas13(Rx).^[^ ^80^ ^]^ Methyltransferase *GhMTA* and demethylase *GhALKBH10* were cloned and fused to the C‐terminus of dCas13(Rx) to construct the vectors pRGEB32‐GhU6.7‐dCas13(Rx)‐GhMTA and pRGEB32‐GhU6.7‐dCas13(Rx)‐GhALKBH10, respectively. For subcellular localization analysis in tobacco, we cloned dCas13(Rx) with a GFP‐tag into the 1300–35S‐GFP plasmid backbone to generate 35S‐dCas13(Rx)‐GFP as a control. The 35S‐dCas13(Rx)‐GhMTA‐GFP and 35S‐dCas13(Rx)‐GhALKBH10‐GFP vectors were constructed by homologous recombination. All primers and synthesized sequences used in this study were listed in Table S3, Supporting Information.

### Western Blot

The samples collected from tobacco leaves 3 days after transformation were ground into a fine powder using liquid nitrogen and homogenized in a lysis buffer (50 mm Tris‐HCl, 150 mm NaCl, 5 mm MgCl_2_, 10% glycerol, 0.1% NP‐40, 0.5 mm DTT, 1 mm PMSF, and 1x Protease Inhibitor Cocktail). The leaf lysates were incubated on ice for 30 min and then centrifuged at 12 000 × g at 4 °C for 30 min. The supernatant was extracted, mixed with 5× SDS loading buffer, and heated at 98 °C for 10 min. The protein mixtures were loaded onto a 4–12% polyacrylamide gel for electrophoresis (PAGE) and subjected to electrophoresis at a constant voltage of 120 V until the front dye reached the bottom of the gel. Subsequently, the protein samples were transferred to 0.45 µm PVDF membranes (Merck) using the Trans‐Blot SD semi‐dry transfer cell (Bio‐Rad) and blocked in 5% nonfat milk in TBST. The membranes were then incubated with anti‐GFP and goat anti‐mouse IgG antibodies (Abclonal, China) for Western blotting, following the manufacturer's instructions.

### Design of gRNAs Targeting *GhECA1* and *GhDi19*

The specific m^6^A sites in *GhECA1* and *GhDi19* transcripts were selected to design gRNAs. All gRNAs were designed using the Cas13 design tool (https://cas13design.nygenome.org/) and selected for high specificity. Subsequently, NCBI (https://blast.ncbi.nlm.nih.gov/Blast.cgi) was used to screen the potential off‐target transcripts in the cotton genome. A previous study had shown that utilizing the tRNA‐gRNA transcription unit effectively enhances gRNA transcript levels in the CRISPR/Cas system for plant genome editing.^[^ ^66^, ^68^, ^69^ ^]^ The p*GhU6*‐7 was selected to drive the gRNAs transcription unit. All sequences of gRNAs were provided in Table S1, Supporting Information.

### Plant Transformation

All constructs were introduced into *Agrobacterium* tumefaciens strain GV3101. For the transient transformation of *Nicotiana benthamiana* leaves, strains containing various expression constructs were resuspended and adjusted to OD600 = 1.0 in an activation solution (110 mm MES pH 5.6, 10 mm MgCl_2_, and 150 µm acetosyringone), followed by a 3 h incubation period. Subsequently, the activated strains were combined in ratios of 1:1 or 1:1:1 with the pSou‐p19 strain, infiltrated into 4‐week‐old tobacco leaves, and grown under standard conditions for 72 h before observation.

For the transformation of cotton, the upland cotton (*Gossypium hirsutum*) cultivar JIN668 was used as the transformation recipient.^[^ ^81^, ^82^ ^]^ Seeds were sterilized and cultured in a dark chamber for 6–7 days at 30 °C. Hypocotyls were cut into 5–7 mm segments and used as explants for *Agrobacterium*‐mediated transformation, following the previous reports.^[^ ^83^, ^84^ ^]^

### Molecular Analyses of Transgenic Plants

Genomic DNA was extracted using the CTAB method, and positive transformants were identified through PCR with dCas13(Rx)‐specific primers. Total RNA was extracted and reverse‐transcribed into cDNA using the polysaccharide polyphenol RNA Extraction Kit (Tiangen, China). For each sample, 1 µg of total RNA was transcribed into cDNA using M‐MLV reverse transcriptase (Promega, USA). qRT‐PCR was performed in a CFX96 real‐time PCR system using SYBR Green Supermix (Bio‐Rad Laboratories, CA, USA). The thermal cycling parameters were as follows: 95 °C for 2 min, followed by 40 cycles of 95 °C for 15 sec and 60 °C for 35 sec. Empty vector‐transformed plants were used as the negative control, and *GhUBQ7* was used as the internal reference gene to normalize the transcript levels of target genes. All assays were repeated in three independent experiments. The primers used were listed in Table S3, Supporting Information.

### Parallel Reaction Monitoring (PRM)

Protein Extraction: The extraction of protein was carried out following a method with some modifications.^[^ ^85^, ^86^ ^]^ Trifoliate leaves from transgenic and wild‐type plants at the same stage were collected. The samples were pulverized into a fine powder using liquid nitrogen. The powder was then mixed with the lysis buffer at a ratio of 1:10 (weight to volume) and thoroughly vortexed. The mixture was homogenized and incubated on ice for 30 min, followed by centrifugation at 13 000 rpm and 4 °C for 15 min. Pre‐chilled acetone was added at a 1:5 volume ratio. The sample was centrifuged at 5 000 rpm and 4 °C for 5 min, and then rinsed with pre‐chilled acetone three times. Lysis buffer was added at a 1:5 (weight to volume) ratio to the protein powder. Vortexing was done to completely dissolve the sample, followed by centrifugation at 13 000 rpm and 4 °C for 15 min.

Membrane‐assisted Protein Digestion: Equal amounts of protein from each sample were placed in a 10 kDa ultrafiltration tube. A 40:1 ratio of solution volume to 1 M DTT volume was prepared, and the mixture was then incubated in a 37 °C water bath for 1 h. After reducing the solution volume by 20:1, the mixture was agitated and left in the dark at room temperature for 30 min. Subsequently, 300 µL of 25 mm ammonium bicarbonate was added, and the mixture was centrifuged at 12 000 rpm (13 400 g) for 10 min. This process was repeated three times. Trypsin was added to the ultrafiltration tube at a ratio of 1:50 protein mass, and the reaction was allowed to proceed overnight at 37 °C. The next day, 100 µL of 25 mm ammonium bicarbonate was added and centrifuged at 12 000 rpm for 10 min. The process was repeated three times, and the digested peptide solution was collected and incubated at 37 °C overnight. The following day, another 100 µL of 25 mm ammonium bicarbonate was added, and the mixture was centrifuged at 12 000 rpm for 10 min.

Ziptip C18 Solid Phase Extraction: The equilibration of the C18 solid‐phase extraction column involves aspirating 10 µL of a solution containing 2% acetonitrile (ACN) and 0.1% formic acid (FA). Sample loading involves repeatedly aspirating and evaporating the enzyme‐digested peptide solution at least 10 times. The desalting wash involves aspirating 10 µL of 2% ACN with 0.1% FA, and this process was repeated five times. Elution includes aspirating 10 µL of 50% ACN with 0.1% FA, followed by evaporating it 10 times consecutively. The eluate was collected in an EP tube and then transferred to a rotary vacuum concentrator for drying under vacuum.

LC‐MS/MS and Analysis: The peptide mixture was dissolved in 10 µL of a 0.1% formic acid (FA) solution and then separated using a Vanquish liquid chromatography system and a nanoViper C18 chromatographic column (75 µm × 250 mm, 2 µm). The mobile phase A consisted of 0.1% FA in water, while mobile phase B comprised 80% acetonitrile with 0.1% FA in water. The elution gradient ranged from 4% to 60%, with a total elution time of 66 minutes and a flow rate of 0.6 µL mi^−1^n. The peptide mixture was analyzed using an Orbitrap Q‐Exactive‐HF mass spectrometer, and the resulting mass spectrometry data were analyzed using MaxQuant (version 2.0.1.0) and fasta‐4.fasta.

### SELECT Technology for the Detection of m^6^A Levels

Detection of m^6^A levels at targeted sites was based on the SELECT technology modified from a previous protocol.^[^ ^70^ ^]^ Total RNAs were quantified using the polysaccharide polyphenol RNA Extraction Kit (Tiangen, China). Total RNA (1500 ng) was mixed with 40 nm up the probe, 40 nm down the probe, and 5 µm dNTP in 17 µL of 1× CutSmart buffer (NEB). The RNA and primers were incubated at a temperature gradient: 90 °C for 1 min, 80 °C for 1 min, 70 °C for 1 min, 60 °C for 1 min, 50 °C for 1 min, and 40 °C for 6 min, 4 °C hold. The RNA and primer mixture were incubated with 3 µL of 0.01 U Bst 2.0 DNA polymerase, 0.5 U SplintR ligase, and 10 nm ATP at 40 °C for 20 min, and then denatured at 80 °C for 20 min. Subsequently, a 20 µL qPCR reaction was set up, containing 2 µL of the final reaction mixture, 200 nm SELECT primers, and 1× SYBR Green Master Mix (Bio‐Rad Laboratories, CA, USA). SELECT qPCR was performed with the following program: 95 °C for 2 min; 95 °C for 15 s, then 60 °C for 35 s for 40 cycles; 95 °C for 15 s; 60 °C for 1 min; 95 °C for 15 s; 4 °C hold. Ct values of the samples were normalized to their corresponding Ct values of the control. All assays were conducted in three independent experiments. The primers and probes used in the SELECT assays were listed in Table S3, Supporting Information.

### Drought Treatments

Soil Drought: Prepare a nutrient‐rich soil mixture by combining vermiculite and substrate in a 2:1 ratio. Select genetically modified material seeds and wild‐type seeds, then soak them for germination one day before planting. Plant the seeds in the soil within a well‐lit cultivation room until the seedlings reach a uniform growth stage. Water the plants the night before the drought treatment and remove any excess water the following morning to allow them to dry out naturally.

Hydroponic Drought: Select seeds with high viability and plant them in vermiculite. After the seedlings have grown for 4–5 days in the illuminated cultivation chamber, rinse the vermiculite with water and choose seedlings that show consistent growth for hydroponic cultivation. During the hydroponic process, a modified Hoagland nutrient solution was used for cultivation, following a specific formula: 2.5 mm KNO_3_, 2.5 mm Ca(NO_3_)_2_, 0.5 mm KH_2_PO_4_, 1 mm MgSO_4_, 0.5 mm KOH, 0.15 mm EDTA‐FeSO_4_, 15 µm H_3_BO_3_, 15 µm MnSO_4_, 4.5 µm ZnSO_4_, 0.015 µm CuSO_4_, 0.15 µm Na_2_MoO_4_, 0.75 µm KI, 0.015 µm CoCl_2_. Once the seedlings reach the two‐leaf stage, simulate drought treatment by using 15% polyethylene glycol 6000 (PEG‐6000), while the control group continues to receive the standard nutrient solution. Contents of malondialdehyde (MDA) and proline (Pro) were measured using kits provided by Grace Biotechnology Co., Ltd. (Suzhou, China). 4–5 seedlings were pooled for each biological replicate, and at least three biological replicates were measured in the experiments. Statistical significance was determined using a two‐sided t‐test on three biological replicates: *p < 0.05, **p < 0.01.

### Transcription Inhibition Assay

The procedure was based on the method described previously with minor modifications.^[^ ^42^ ^]^ Four‐week‐old cotton leaves from the wild type (JIN668) and transgenic plants were cut into leaf discs. These leaf discs were immersed in an actinomycin D solution (AbMole, M4881) with a final concentration of 20 ug mL^−1^. After infiltration for 1 h, five‐leaf discs were collected and marked as time 0 controls. Subsequent samples were harvested every 2 h in triplicate. qRT‐PCR was performed using the ABI 7500 system (Applied Biosystems, Foster City, CA) to determine mRNA expression levels, with the internal reference gene *GhUBQ7*. The 2^−ΔCt^ method was used to present relative changes in gene expression levels (Schmittgen and Livak, 2008). The mRNA degradation was calculated as the percentage of gene expression at the sampled time relative to the control. Primers were listed in Table S3, Supporting Information.

### MeRIP‐seq

MeRIP‐seq was conducted based on previously described protocols with minor modifications.^[^ ^87^, ^88^ ^]^ Total RNA was extracted from cotton leaves, and 100 µg of total RNA was subjected to polyA selection to isolate 2 µg of intact mRNA using a Dynabeads mRNA Purification Kit (Invitrogen). All isolated mRNA was fragmented and then incubated with an m^6^A antibody (NEB, USA) for immunoprecipitation. Both input and IP RNA samples were used for RNA library construction with the NEBNext Ultra II Directional RNA Library Prep Kit for Illumina (New England Biolabs), following the manufacturer's instructions. Subsequently, all RNA libraries were sequenced through high‐throughput sequencing (CloudSeq Biotech, China). Three independent MeRIP‐seq experiments were performed for each line.

### ATAC‐seq

ATAC sequencing was conducted on cotton leaf cells as described previously.^[^ ^89^ ^]^ Freshly prepared nuclei without formaldehyde fixation were directly used for ATAC‐seq. In brief, the nuclei were washed twice in 1× PBS solution containing 1% (v/v) Triton X‐100 to eliminate plant organelle debris after tagmentation. Subsequently, DNA purification was carried out using the Minelute PCR purification kit (Qiagen). After PCR amplification using index primers that match the Nextra adapter (Illumina, https://emea.illumina.com), the ATAC‐seq libraries containing a DNA insert between 50 and 150 bp were gel‐purified. The cleaned‐up libraries were quantified and pooled for sequencing (Frasergene, China). Two independent ATAC‐seq experiments were performed for each line.

### RNA‐seq

RNAs were extracted using the above method, fragmented, and reverse‐transcribed to cDNAs with a HiScript II One‐Step RT‐PCR Kit following the manufacturer's protocol. An RNA‐seq library was prepared with a TruSeq Stranded Total RNA Library Preparation Kit using the standard protocol. The transcriptome libraries were sequenced on a 150‐bp paired‐end Illumina Xten platform. Three independent RNA‐seq experiments were performed for each line.

## Conflict Of Interest

The authors have no conflict of interest to declare.

## Author Contributions

J. S., Z. X., Y. X., and N. X., conceived and designed the experiments. L.Y. performed the experiments and wrote the manuscript. M. A., L. B., A. H., Z. H., et al. participated in the experiments. All authors have read and approved the final manuscript.

## Supporting information

Supporting Information

Supplemental Table 1

## Data Availability

The data that support the findings of this study are available in the supplementary material of this article.
