# Supplementary material for: CRISPR/dCas13(Rx) Derived RNA N6‐methyladenosine (m6A) Dynamic Modification in Plant
Source: Adv Sci (Weinh). 2024 Sep 4;11(39):2401118. doi: 10.1002/advs.202401118 (PMC11497087; doi:10.1002/advs.202401118)
Supplement: Supplementary file 1 — Supporting Information [file ADVS-11-2401118-s001.pdf]

ADVANCED SCIENCE
Open Access

Supporting Information

for *Adv. Sci.*, DOI 10.1002/advs.202401118

CRISPR/dCas13(Rx) Derived RNA N^6-methyladenosine (m^6A) Dynamic Modification in Plant

Lu Yu, Muna Alariqi, Baoqi Li, Amjad Hussain, Huifang Zhou, Qiongqiong Wang, Fuqiu Wang, Guanying Wang, Xiangqian Zhu, Fengjiao Hui, Xiyan Yang, Xinhui Nie, Xianlong Zhang and Shuangxia Jin**

Supplementary Data

Supplementary Figures

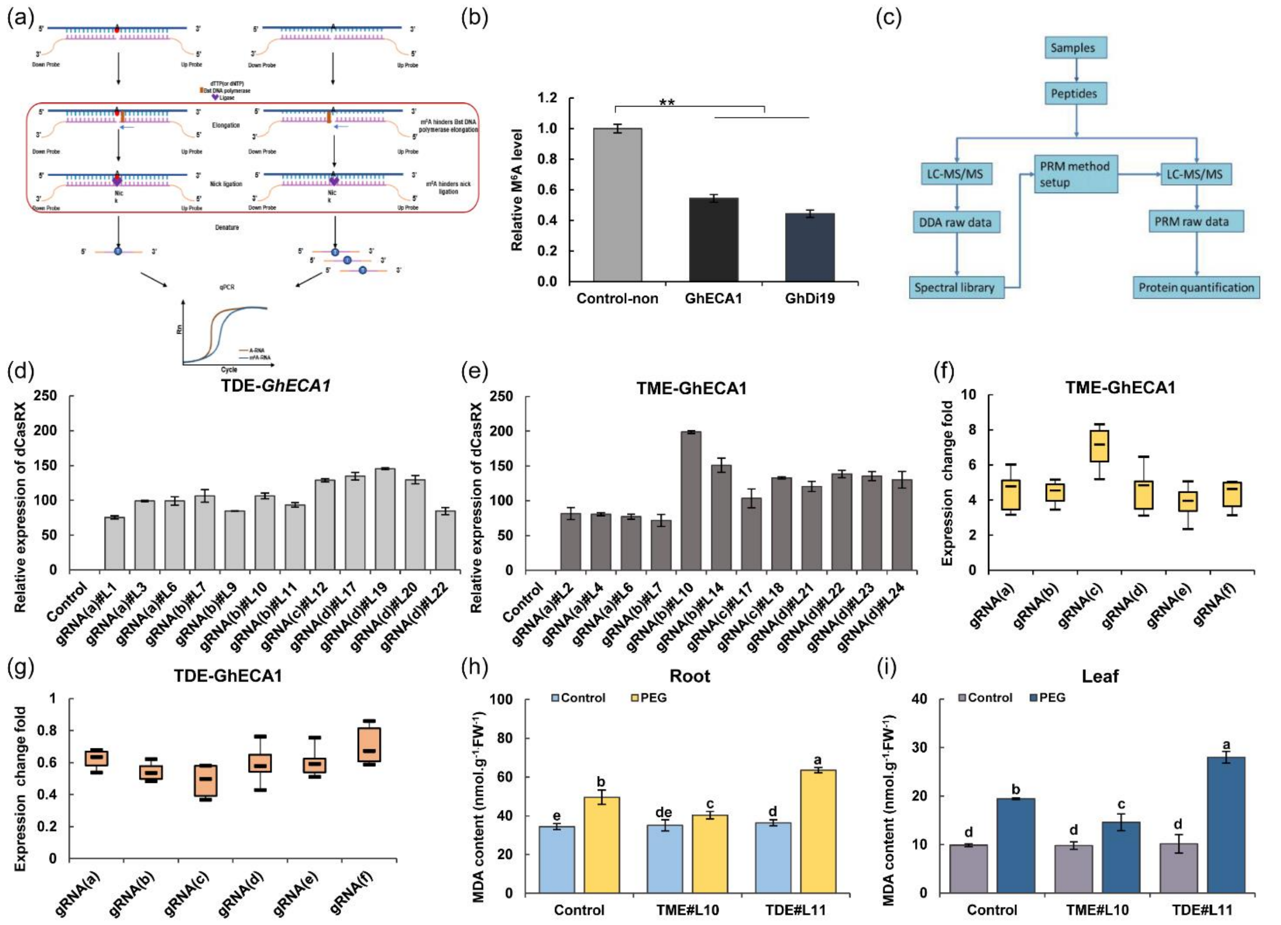

Supplemental Figure S1. (a) Schematic diagram of the site-specific m^{6}A detection method termed SELECT. **(b)** Relative m^{6}A enrichment level of *GhECA1* and *GhDi19* transcripts that nearby nucleotides did not show any m^{6}A modification. **P < 0.01. **(c)** Technology roadmap of PRM method. **(d)** Relative transcript levels of *dCas13(Rx)* gene in T1 plants of TDE-edited *GhECA1*. **(e)** Relative transcript levels of *dCas13(Rx)* gene in T1 plants of TME-edited *GhECA1*. **(f)** Statistical analysis of the relative gene transcript levels in TME-edited *GhECA1* plants for each gRNA. **(g)** Statistical analysis of the relative gene transcript levels in TDE-edited *GhECA1* plants for each gRNA. **(h)** MDA content in the roots of the 4-week-old transgenic seedlings treated with 15% PEG-6000 after 36 h. **(i)** MDA content in the leaves of the 4-week-old transgenic seedlings treated with 15% PEG-6000 after 36 h. Error bars are given as the mean ± S.D. (n = 3). Statistical significance is denoted by different letters with P < 0.05 using Student's t-test.

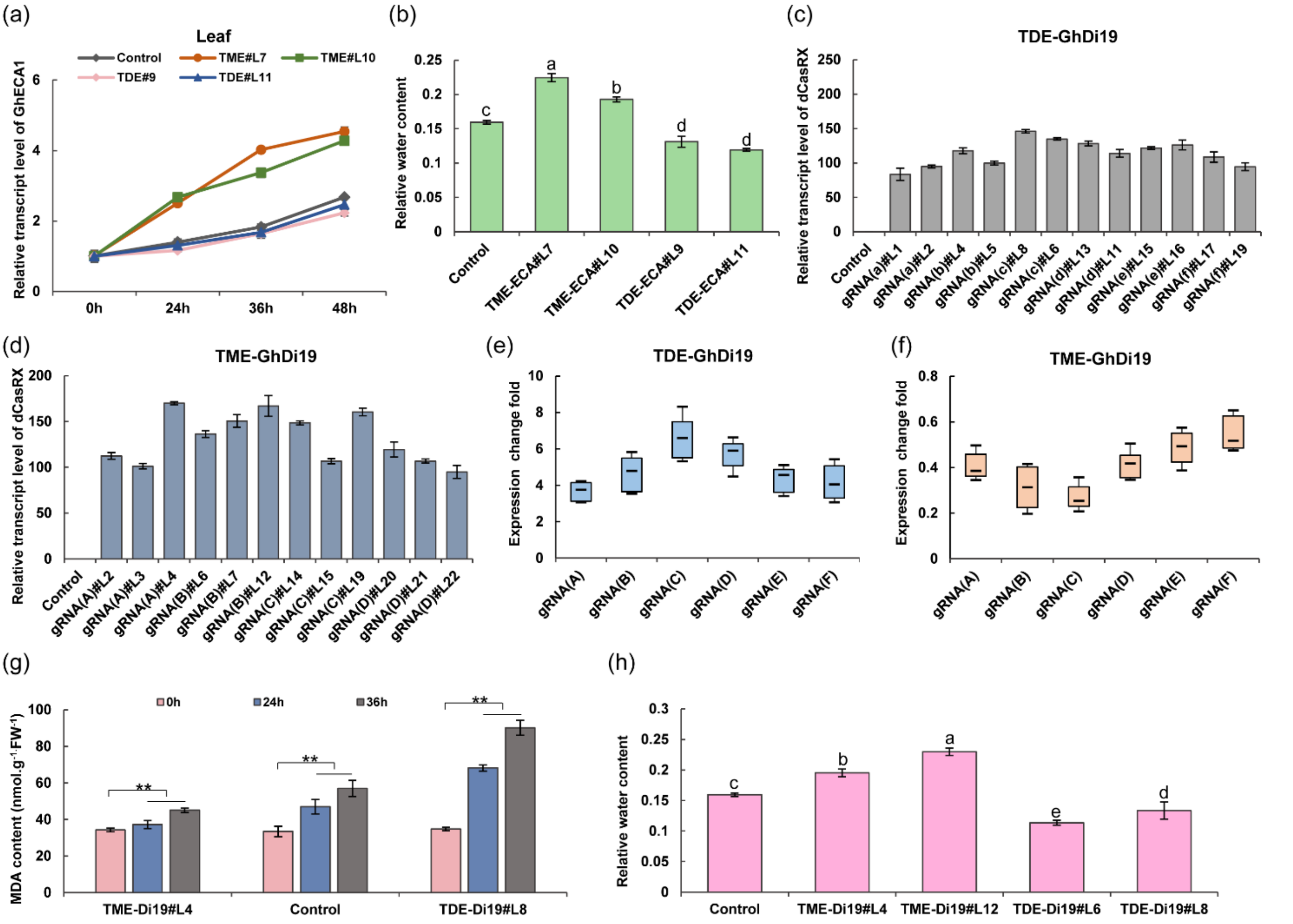

Supplemental Figure S2. (a) Relative transcript levels of *GhECA1* in the leaves of the 4-week-old transgenic seedlings treated with 15% PEG-6000. Mean ± S.D. (n = 3). **(b)** Relative water content in the leaves of TME-edited *GhECA1*, TDE-edited *GhECA1*, and wild-type plants. Error bars are given as the Mean ± S.D. (n = 3). Statistical significance is indicated by different letters with P < 0.05 using Student's t-test. **(c)** Relative transcript levels of *dCas13(Rx)* gene in TDE-edited *GhDi19* T1 plants. **(d)** Relative transcript levels of *dCas13(Rx*) gene in TME-edited *GhDi19* T1 plants. Mean ± S.D. (n = 3). **(e)** Statistical analysis of the relative gene transcript levels in TDE-edited *GhDi19* plants for each gRNA. **(f)** Statistical analysis of the relative gene transcript levels in TME-edited *GhDi19* plants for each gRNA. (**g**) MDA content in the leaves of the 4-week-old transgenic seedlings treated with 15% PEG-6000 for 36 h. Mean ± S.D. (n = 3). **(h)** Relative water content in the leaves of TME-edited *GhDi19*, TDE-edited *GhDi19*, and wild-type plants. Error bars are presented as the mean ± S.D. (n = 3). Statistical significance is indicated by different letters with P < 0.05 using Student's t-test.

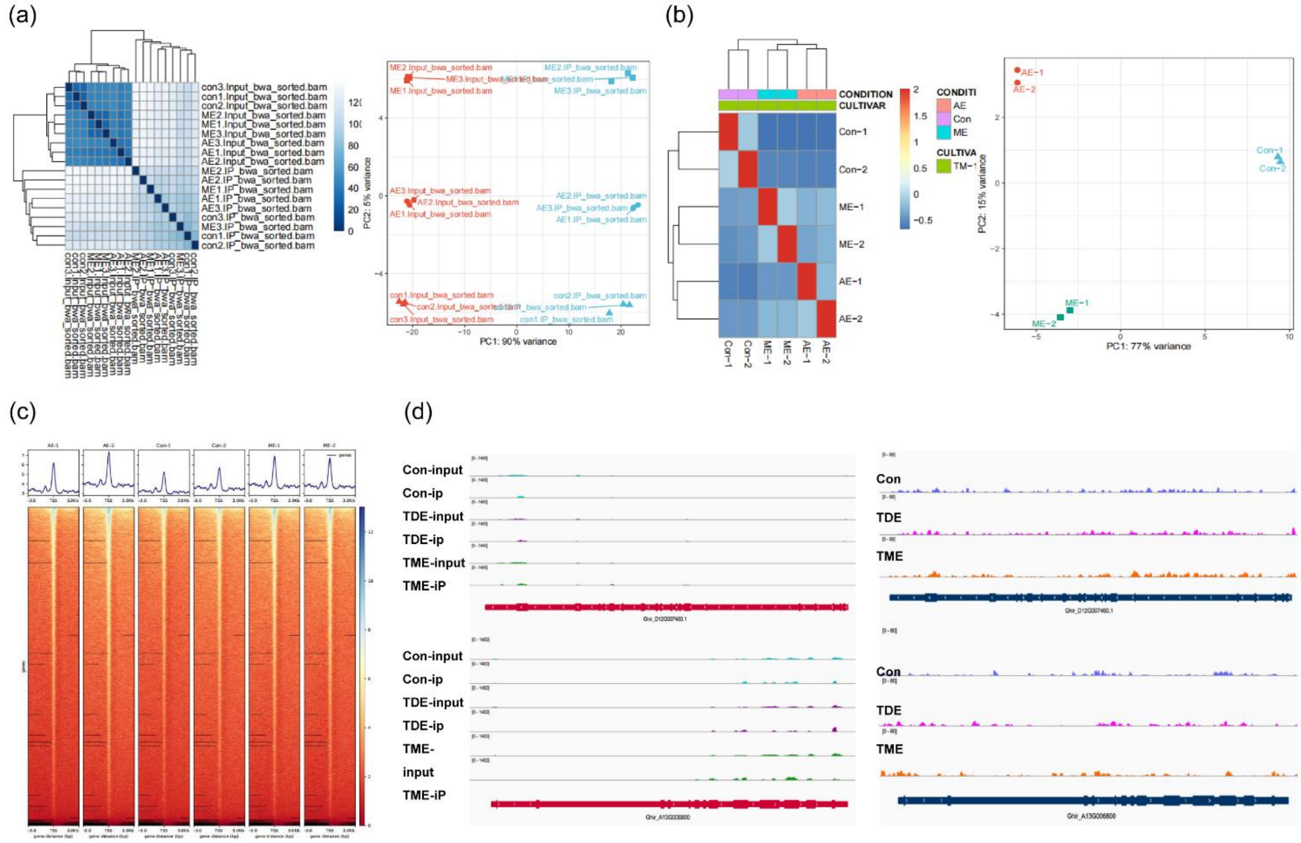

Supplemental Figure S3. (a) Correlation heatmap and PCA analysis of MeRIP-seq samples. MeRIP-seq analysis was performed with three independent biological replicates. **(b)** Correlation heatmap and PCA analysis of ATAC-seq samples. ATAC-seq analysis was performed with two independent biological replicates. **(c)** Distribution of partial samples of ATAC signals in the TSS region. **(d)** Genome browser view of m^{6}A peak and genome browser tracks of ATAC-seq libraries on two other transcripts with the high similarity to the target sequence.

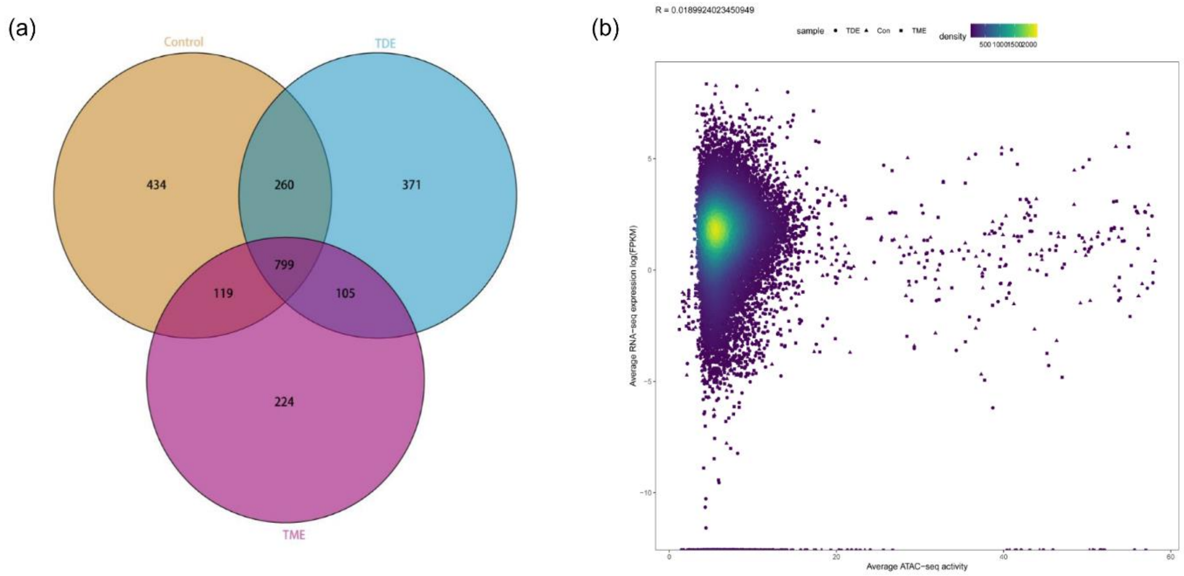

Supplemental Figure S4. (a) Venn plots showing overlapped m^{6}A peaks among Control, TDE-*GhECA1*, and TME-*GhECA1*. MeRIP-seq analysis was performed with three independent biological replicates. **(b)** Scatter plot of the correlation between chromatin accessibility and gene expression in m^{6}A-editing lines of Control, TDE-*GhECA1*, and TME-*GhECA1*. Control: wild-type plant. RNA-seq analysis was performed with three independent biological replicates. ATAC-seq analysis was performed with two independent biological replicates.

Supplementary Tables

Supplemental Table S1. Target gRNAs of *GhECA1* and *GhDi19*.

gene	Name	Target site sequence
GhECA1	sgRNA-a	AACACAGATTGTAAAAACGCCAT
	sgRNA-b	ACACAGATTGTAAAAACGCCATG
	sgRNA-c	ACAGATTGTAAAAACGCCATGTT
	sgRNA-d	AAAACGCCATGTTAGAGGACAGT
	sgRNA-e	CATGTTAGAGGACAGTTCCCAAG
	sgRNA-f	ATTTTAATCGTTTTAGAGGCTAG
GhDi19	sgRNA-A	ATAAATTTTCAACCCAAAAGGCG
	sgRNA-B	TAAATTTTCAACCCAAAAGGCGA
	sgRNA-C	TTTTCAACCCAAAAGGCGAAAAA

sgRNA-D	TTCAACCCAAAAGGCGAAAAAGG
sgRNA-E	CCCAAAAGGCGAAAAAGGAAGAT
sgRNA-F	GAAGATTTTAATTTGAGGGGTTA

Supplemental Table S2. The number of transgenic plants.

Gene Target	sgRNA(n)	Number of checks	T0 positive strains	Number of edited plants	T1 positive strains	Transgenic positive efficiency（%）
TDE-edited *GhECA1*	sgRNA-a	12	7	5	3	58.3
	sgRNA-b	10	7	3	3	70
	sgRNA-c	8	5	2	2	62.5
	sgRNA-d	10	6	4	4	60
	sgRNA-e	11	9	5	3	81.8
	sgRNA-f	6	4	3	3	66.6
TME-edited *GhECA1*	sgRNA-a	9	5	4	3	55.6
	sgRNA-*b*	11	6	4	3	54.5
	sgRNA-c	14	9	7	3	64.3
	sgRNA-d	9	5	4	2	55.6
	sgRNA-e	11	6	3	2	54.5
	sgRNA-f	8	5	3	3	62.5
TDE-edited *GhDi19*	sgRNA-A	9	5	2	2	55.6
	sgRNA-B	8	6	3	2	75
	sgRNA-C	12	6	5	3	50
	sgRNA-D	16	7	4	2	43.8
	sgRNA-E	11	5	2	1	54.5
	sgRNA-F	12	5	3	1	41.7
TME-edited *GhDi19*	sgRNA-A	8	4	3	2	50
	sgRNA-B	9	4	3	1	44.5
	sgRNA-C	15	11	7	2	73.3
	sgRNA-D	11	7	4	2	63.6
	sgRNA-E	16	7	3	1	43.8
	sgRNA-F	16	8	3	2	50

Supplemental Table S3. The primers used in the study.

Primers	Sequences（5'-3'）
dCas13(Rx)-1F	GCAAAGGGTATGGGAGTGA
dCas13(Rx)-1R	AACCCAATGAGCAAGACCA
dCas13(Rx)-2F	GCAAAGGGTATGGGAGTGA
dCas13(Rx)-2R	AACCCAATGAGCAAGACCA
p*GhU6*-F	TGTGCCACTCCAAAGACATCAG
p*Ubi*-R	TGTTGGTCGCCGTTAGGA
qrtdCas13(Rx)-1F	AAGGAAATGGCTGAGAGGGC
qrtdCas13(Rx)-1R	AGCGAACAAGGTGCATGTCT
qrtdCas13(Rx)-2F	TCGCAAAGGGTATGGGAGTG
qrtdCas13(Rx)-2R	TCCGATCTTGTAACCAGCGT
qrt *GhDi19*-1F	TCGACATCGTTGGCTTGTGT
qrt*GhDi19*-1R	ATGCGCAACCATATCAACGC
qrt*GhECA1*-1F	AGTTGAGAATCGGCGCAAGA
qrt*GhECA1*-2R	GCCGCCAGCAATAAGATTCG
qrtdCas13(Rx)-1F	ACCAAATTGTGCGCTGGAAT
qrtdCas13(Rx)-1R	AGCTTTGGGTTAGCAGACTCC
select-F	ATGCAGCGACTCAGCCTCTG
select-R	TAGCCAGTACCGTAGTGCGTG
select-*GhECA1*_up	tagccagtaccgtagtgcgtgGGGGTTACAGATGAAACCAATAACACATTA
select-*GhECA1*_down	5phos/ATAACACAGATTGTAAAAACGCCATGTTAGcagaggctgagtcgctgcat
select-*GhDi19*_up	tagccagtaccgtagtgcgtgCCATTGTTTCTAAATCACCCTCTTGCTTA
select-*GhDi19*_down	5phos/ATAAATTTTCAACCCAAAAGGCGAAcagaggctgagtcgctgcat
UBQ7-F	GAAGGCATTCCACCTGACCAAC
UBQ7-R	CTTGACCTTCTTCTTCTTGTGCTTG
qrt*GhALKBH10*-1F	GGCTACGAGGCACTGGATAC
qrt*GhALKBH10*-1R	GGCAAGAAGACCCCTGTACC
qrt*GhALKBH10*-2F	GAAGAATGTGATGCACGCCC

qrt*GhALKBH10*-2R	CAAGTCACCCAGCTTAGCCA
qrt*GhMTA*-1F	GGTTACAAGCGGTGTGAGGA
qrt*GhMTA*-1R	TCGAACCTCGGCAACAATGA
qrt*GhMTA*-2F	CGCAACCTTAACGTTCCTGC
qrt*GhMTA*-2R	TCCTCACACCGCTTGTAACC
GhECA1-R	TTTTATTCCGCTATCTGG
GhECA1-F	GGGTATGTGACAAGGGTG
GhDi19-R	GGCGTTGATATGGTTG
GhDi19-R	GAAAGCGTGGAATGAG

Supplementary Sequences

> dCas13(Rx) (codon optimization sequence)

GGGAGTGAAGCCAGTATTGAAAAGAAGAAATCATTCGCAAAGGGTATGGGAGTGAAAAGTACATTGG

TTAGCGGTAGTAAGGTTTATATGACTACATTTGCTGAAGGTTCAGATGCTAGACTTGAGAAGATTGTTGA

GGGTGATTCTATCAGGTCAGTTAATGAGGGAGAAGCTTTCTCAGCTGAGATGGCTGATAAGAACGCTG

GTTACAAGATCGGAAACGCTAAGTTCTCTCATCCTAAAGGATACGCTGTTGTTGCTAATAACCCTCTTTA

TACTGGTCCAGTTCAACAAGATATGCTTGGATTGAAGGAGACATTGGAAAAAAGATACTTCGGTGAATC

TGCTGATGGAAACGATAACATCTGTATCCAAGTTATCCATAACATCTTGGATATCGAGAAGATCCTTGCT

GAATACATTACTAATGCTGCTTATGCTGTTAATAACATCTCAGGTTTGGATAAGGATATCATCGGTTTCGG

AAAGTTCTCTACTGTTTACACATACGATGAGTTCAAGGATCCTGAACATCATAGGGCTGCTTTCAACAA

CAACGATAAGCTTATCAACGCTATCAAAGCTCAATACGATGAGTTCGATAACTTTTTGGATAATCCAAGA

CTTGGTTACTTTGGACAAGCTTTCTTTTCAAAGGAAGGTAGAAACTACATCATCAACTACGGAAACGA

GTGCTATGATATCTTGGCTCTTTTGTCTGGTCTTGCTCATTGGGTTGTTGCTAACAATGAAGAGGAATCT

AGAATTTCAAGGACATGGCTTTACAACTTGGATAAGAACCTTGATAACGAATACATCTCAACCCTTAAC

TACTTGTACGATAGAATCACCAACGAGTTGACTAATTCTTTCTCAAAAAACTCTGCTGCTAACGTTAACT

ACATTGCTGAAACCCTTGGTATCAATCCAGCTGAGTTCGCTGAACAATACTTCAGGTTCTCTATCATGAA

GGAGCAAAAGAATCTTGGATTCAACATCACTAAGTTGAGAGAAGTTATGCTTGATAGGAAGGATATGTC

AGAGATTAGAAAGAACCATAAGGTTTTCGATTCTATCAGGACAAAGGTTTACACCATGATGGATTTCGT

TATCTACAGATACTACATCGAGGAAGATGCTAAGGTTGCTGCTGCTAACAAATCATTGCCTGATAACGA

GAAGTCTCTTTCAGAAAAGGATATCTTCGTTATCAACTTGAGGGGTTCTTTCAACGATGATCAAAAGGA

TGCTCTTTATTACGATGAAGCTAACAGAATTTGGAGGAAGTTGGAGAACATCATGCATAACATCAAGGA

ATTCAGAGGAAACAAGACAAGGGAGTACAAGAAAAAGGATGCTCCTAGACTTCCAAGGATTTTGCCA

GCTGGTAGAGATGTTTCTGCTTTCTCAAAGCTTATGTACGCTTTGACCATGTTTCTTGATGGAAAAGAG

ATCAACGATCTTTTGACCACTCTTATCAACAAGTTCGATAACATCCAATCATTTTTGAAGGTTATGCCTC

TTATCGGTGTTAATGCTAAGTTCGTTGAGGAATATGCTTTCTTTAAGGATTCAGCTAAGATCGCTGATGA

ACTTAGATTGATCAAGTCTTTCGCTAGGATGGGAGAGCCAATTGCTGATGCTAGAAGGGCTATGTATATC

GATGCTATTAGAATCTTGGGAACCAACCTTTCATACGATGAACTTAAAGCTTTGGCTGATACTTTTTCTT

TGGATGAGAACGGAAACAAGCTTAAAAAGGGAAAGCATGGAATGAGGAACTTCATCATCAACAACGT

TATTTCTAATAAGAGATTCCATTACTTGATCAGATATGGTGATCCTGCTCATCTTCATGAGATTGCTAAGA

ACGAAGCTGTTGTTAAGTTCGTTTTGGGAAGAATTGCTGATATCCAAAAGAAGCAAGGTCAAAACGGA

AAGAACCAAATCGATAGGTACTACGAAACTTGTATCGGTAAAGATAAGGGAAAATCTGTTTCAGAGAA

GGTTGATGCTCTTACTAAGATCATCACTGGTATGAACTACGATCAATTCGATAAAAAGAGGTCTGTTATT

GAGGATACTGGAAGAGAAAATGCTGAGAGGGAAAAGTTTAAAAAGATTATCTCTCTTTACTTGACAGT

TATCTACCATATCTTGAAGAACATCGTTAACATCAACGCTAGATACGTTATCGGTTTCCATTGTGTTGAAA

GGGATGCTCAACTTTACAAGGAGAAGGGATACGATATCAACCTTAAAAAGTTGGAGGAAAAGGGTTTT

TCTTCAGTTACCAAATTGTGCGCTGGAATTGATGAAACTGCTCCTGATAAGAGAAAAGATGTTGAGAA

GGAAATGGCTGAGAGGGCTAAAGAATCTATTGATTCATTGGAGTCTGCTAACCCAAAGCTTTATGCTAA

CTACATCAAGTACTCAGATGAAAAGAAGGCTGAGGAATTTACAAGACAAATTAACAGGGAGAAGGCTA

AGACAGCTTTGAATGCTTATCTTAGAAACACCAAGTGGAATGTTATTATCAGAGAAGATCTTTTGAGGA

TTGATAACAAGACATGCACCTTGTTCGCTAATAAAGCTGTTGCTCTTGAAGTTGCTAGATACGTTCATGC

TTATATTAACGATATCGCTGAGGTTAACTCATACTTCCAACTTTACCATTACATTATGCAAAGAATTATCAT

GAACGAGAGATACGAAAAGTCTTCAGGTAAAGTTTCTGAATACTTCGATGCTGTTAACGATGAGAAAA

AGTACAACGATAGACTTTTGAAGCTTTTGTGTGTTCCTTTCGGATACTGCATCCCAAGATTCAAGAACT

TGTCTATCGAAGCTCTTTTCGATAGGAATGAGGCTGCTAAGTTTGATAAGGAGAAGAAGAAGGTGTCTG

GTAACAGTGGTAGTGGG

> The sequence of gene *GhALKBH10*

ATGCCAATGGCGGCGGGGGCAGCGACGCCGAGGGAAAGGGTTCAAGCTATGGGGCCAGCAGCGGTGG

TACCGATGATGCAAGCTGTGCCGGCTGTTGCTGATGTTTTGGCCAAGGACACGATCATTTCTTGGTTCC

GAGGGGAATTCGCGGCGGCCAATGCCATCATCGATGCTCTTTGCGGTCATTTGGCGCAGCTTCAAGGA

GGGGGCGGTGAAGGATCCGAGTACGAGGCGGTGTTCGCGGCGATCCACAGGAGGCGCTTGAATTGGA

TTCCTGTGTTGCAGATGCAGAAGTACCACTCCATTGCTGATGTGACGGCGGAGCTGAAAAAGGTGACT

GCCAAGAAGACCGGAGGAGGAGATGGAATTGGCAAAGAGGAAATGGAAGGTGGCGTTGGCGGAGGA

GGAGAAGACGGCGGTTGTTTGGATGATGTGAAAGAAGAAGAGAAAGTCGCCGAGGAAGTGGTGGAA

AATGAAGCAAATGGGGAGGTTGGTTGCGAGGAAGAAGAAGATTCCCCTGACAGTGACATTACTGATTC

AGGGTCACAGGAAATTCAACATGTTGAGGAAAACATCGACATTTGTTCTAACCATGAAGAATGTGATG

CACGCCCTTCTCAAATCAAGCTGACGAAAGGTTTTTCTGCCAAGGAACATGTAAAGGGCCACATGGTG

AATGTGGTGAAAGGATTGAAGTTGTATGAGGACGTATTCACCGAGTCGGAGCTGGCTAAGCTGGGTGA

CTTGATGAGTGAACTTCGATCTTCCGGCCAGAATGGGGAATTGTCAGGTGAGACTTTCATTTTATTCAA

CAAACAAATTAAAGGAAACAAGCGAGAGCTGATTCAGTTCGGTGTTCCGATTTTCGGACACATAAAGG

AGGAGCTGACAAGTAACAACCAAACAATCAACATCGAACCAATTCCTACTCTGCTTCAAGATGTCATA

GAGCACTTGATCCAATGGCAACTTATACCTGAATATAAAAAACCAAATGGCTGCATCATTAACTTCTTTG

ATGAGGATGAATATTCACAACCTTTTCTTAAACCACCACATTTGGAGCAACCTATCTCCACTCTTCTCCT

ATCTGAATCAACAATGGCTTTCGGTCGAACTCTTACAAGTGATAGTGAAGGGAACTATAGAGGGCCACT

CCAACTTTCATTGAAAGAAGGGTCTCTTTTAGTTATGAGGGGAAATAGTTCTGACATGGCAAGGCATGT

GATGTGCCCGTCTTCAAACAAGAGGGTCAGCATTACCTTCTTCCGAGTCCGGCCCGATATTAATCAAGG

TCAGTCACCGCCAACTACTCCCCAGTCTGGTGCCATGACTCTATGGCAACCAGGAGTACCAGGTCCATA

TGCAATGTCAAATGGAGTTCTTAGTGGCTACGAGGCACTGGATACAATGCCAAAATGGGGAGTCCTTC

GTGCTCCTGTTGTCATGCTAGCACCTGTGCGCCCAGTGGTAGTGAGCCCCAGAAAACTTCCTCGTGGA

GGTACAGGGGTCTTCTTGCCCTGGACTATGGGATCAAAAAAACACACCAAGCACCTTCCACCACGAGC

CCAGAAAGGAAGAATGCTCGCATTACCTTCTGCTGTCGAAACACATGTATCAGAGTTTACTTCTGAACC

AAGCAACAATCTCAAAGGGAAATCAGAG

> The sequence of gene *GhMTA*

ATGGAGAGCAACTCAGGCGGCGAAGACACCGTAGCAACCATCAAATCAATTCGGACCCAACTCGAAA

CCCGAATCCAAGACCAACATGCCACCCACCTCGACCTCCTTGCCTCCCTCCAAACTCTGGATCCTAACA

TCGTTCCCACCCTCGATCTTTCCCTCCGTTTCGTCTCCGCCTTCAACCGCCGCTCTTTCTCTCCCACTCC

TCCACTCCCCACGCCTAAAAAGATCTCCCACCCGCCCCAACACCCGCCCACCCACTCTGTACCCGATCC

GAAACAGCTCGCCCTTGTTAAACCTGAAGGAGGCGACAAGTTCGCTGACGAGAGCGGCAACCCATTG

TTGATGATGAGAGCCATGGTGGCTGAGTGTTTACTTCAGAGAGTACCGTTTAAAGCGATCGATTCGTCT

ACGGTTTTGAGGAAGTTAGAGAACGACGAGAATATAACTACGGCGGAGAAGGCTGCGATGCGTGAAC

TGGGAGGCGATTCTGGGGCCATTCTCGCGGTGGAAATGGCTTTGAGGTCAATGGCAGAGGATAACGGC

GGCCTTGAGATCGAAGAGTTTGTGGTCGGCGGCAAAAGCAGAGTTATGGTACTTAGCATCGACCGAAC

ACGGTTAGTTCGAGAATTACCCGAAGAACCTCAAAATCATCAAAAAAGAGAAAGGATAAACAACGTTA

ACGAAAGTGAGAACTTGAAAATGAATAGTAACAGCAATAACGAGTGGCTAGCACCGAGACCAATGAG

TGAAATTTGGATGGGAGGGGGAGACCCTGGCATGATGTATCCGCCGGGTGGCCCAATGGCAGGCCCTA

GAGGTTGGGGAATGGGGATGATGGGAAGGCCACCGATGGCACCAAACAGTGGCCTTTTGCCGTCACA

GAGACAGAGTACTGAGGAAGATGATTTGAAGGATTTGGAAGCCTTGTTGAATAAGAAATCGTTTAAGG

AAATGCAGAAATCAAAGACGGGTGAGGAGATTTTGAATATAATTAATCGCCCAACTGCTAGGGAAACA

GCTGTGGCTGCTAAGTTTAAAAGCAAAGGAGGTTCTCAAGTGAGGGAATATTGTTCAGCTTTAACCAA

GGAGGATTGTCGAAGACAATCTGGCTCATTTCTTGCCTGTAAGAAGGTGCATTTTAAGCGGATAATTGC

TCCTCATACAGACATCAGTTTAGGGGATTGTTCATTTCTAGATACCTGCCGGCATATGAAGACATGCAAA

TATGTCCACTACGAGCTTGACCAAACACAAGATGATCTTGGTCCTGAGAAACCTTTGAAGCCTCCACGT

GCTGAGTATTGTTCGGAAGTGGAATTAGGTGAACCACAATGGATTAATTGTGATATCAGGAATTTTAGA

ATGGACATTTTGGGGCAGTTCGGTGTTATTATGGCAGATCCACCATGGGATATTCATATGGAGTTACCTTA

TGGAACAATGGCTGATGATGAGATGCGCAACCTTAACGTTCCTGCATTGCAGACTGATGGTCTGATATT

TCTTTGGGTCACTGGGCGTGCAATGGAGCTAGGACGGGAATGTTTGGAACAATGGGGTTACAAGCGGT

GTGAGGAGATTATTTGGGTGAAAACAAATCAACTTCAGCGAATAATTAGAACAGGAAGAACAGGTCAT

TGGTTAAATCATAGTAAGGAGCATTGCCTTGTAGGAATCAAGGGAAATCCAGAAATAAATAAGAACATT

GATACCGATGTCATTGTTGCCGAGGTTCGAGAGACTAGTCGCAAGCCAGATGAGATGTACCCGATGCTG

GAGAGGATCAGTCCAAGGACAAGGAAGCTTGAACTATTTGCTCGCATGCATAATACTCATGCAGGATG

GATATCGCTTGGGAATCAGTTAAATGGGGTTAGACTGGTTGATGAAGGCCTAAGAGCAAGGTACAAGG

CTGCCTACCCACATGTAGAAGTACAACCTCTCTCTCCTCCCAAAGCTTCTGCCATGGAACTAGACTCTA

CTTCTGCTAGAAGTCCCTTTGCAACAGAGTCAAGATCACAATTTGCGGATCCAGCTGCTCCCGATGCTG

GCCATGCTCCCGAAGAGAGGGCAATGGCTGTAGATACTGATATGACCACC

> The m^{6}A site sequence of gene *GhECA1*

TTAACGAATGGCTGCTGGTTCTGGCCGTCGCATTCCCTGTGATTTTGATCGACGAAGTGTTGAAGTTCG

TAGGGAGGTTGGGACGGCGGATGAGATCATCCAGTCAAAGACCTTTGAAACCCAAAACAGAGTGAAG

ACAGATATGGTATAACTCCGATAAGTGGATACTAGACAGCCAATACTAGCCTCTAAAACGATTAAAATTC

TTGGGAACTGTCCTCTAACATGGCGTTTTTACAATCTGTGT**TATAT**AATGTGTTATTGGTTTCATCTGTA

ACCCCCTCACTTAATTTCAGGAAAAAGGGGGGGGGGAGAACGGTTTGGACCACAACGAACTAGCTA

AGCTAAACTATGCCTTTATTACTTTTGTTTACTAGGTTTTTCATGATTCAAACTCATTTTTTTTTTGTCAGA
AAATGCAAAAGGAAACTTTTCTGAATTAGTAAATTGTTTAGATGTGCAATAACTTTAAATTATCTTATATT
TATTAAAATGTACAACTGTGGATATTTTGAGTACTCATATATAACGACCTTAAATTGAGTTTTAAGTTAAA
TATAATTGGATGTATTGATTTAATTTATATTTTTTATTAGAATT

> The m⁶A site sequence of gene *GhDi19*

TGAACCAATAAATTTTTATAATCTGATTAAGGTAGTGGGAGTATTTTTATATTTATTTTTACAAAATTAGA
AAAATTAAAACGTAAGAAGGCAAAAGAAAATAAGAGTAATATAAAAGGCAAAGACTTGATAGATGAA
ATTTCCTCTTTCGAACGAAATTCACTCTCGAAGAGGGACAACAACAGGCAGCTCTTCTTCCTTTTCTGC
GTCTCATCATCAAACCCCAAACTCTTAACTGGCCTCTTCCTCCTCCTCTTTACTTTAATTTCATTTGCTTT
CACAAATCTAAGACCAGAGCTCCAAAAGATTGAATTTTTAACCCCTCAAATTAAAATCTTCCTTTTTCG
CCTTTTGGGTTGAAAATT**TATAT**AAGCAAGAGGGTGATTTAGAAACAATGGATGCTGATTCATGGAGT
GCTCGTCTTTCTTCAGCTTCTAAGAGATATCAATCGGCTCTTCAATTACGATCTGATATGTTTATGGGGTT
TGAAGAAATTGATGGAGAAGATGAAATAAGAGAGGAGTTTCGATGCCCTTTTTGTTCAGAGTATTTCGA
CATCGTTGGCTTGTGTTGTCACATTGATGATGAGCATCCAGTGGAGGCTAAAAATGGGGTTTGTTCAGT
CTGTGCAGTGAGGGTGGGCGTTGATATGGTTGCGCATATAACCCTACAACATGGAAATATATTTAAGATG
CAGCGCAAGAGGAAATCACGTAAAGGTGGATCTCATTCCACGCTTTCTCTTCTGAGGAAAGAGCTGCG
AGAAGGAAATCTACAGTCCTTCTTCGGGGGTTCTTCTTGTACGGTGTCTTCCAATTCAGCCCCTGATCC
GTTGTTGTCTTCATTTATTTTACCCATGGTTGA
